# Unveiling the functional heterogeneity of cytokine-primed human umbilical cord mesenchymal stem cells through single-cell RNA sequencing

**DOI:** 10.1186/s13578-024-01219-3

**Published:** 2024-03-26

**Authors:** Zhiwei Hu, Duanduan Li, Shiduo Wu, Ke Pei, Zeqin Fu, Yulin Yang, Yinfu Huang, Jian Yang, Chuntao Liu, Junyuan Hu, Cheguo Cai, Yan Liao

**Affiliations:** 1 Shenzhen Beike Biotechnology Co., Ltd, Shenzhen, 518054 China; 2 Shenzhen Beike Biotechnology Research Institute, Shenzhen, 518054 China; 3 Key Laboratory of Systems Health Science of Zhejiang Province, School of Life Science, Hangzhou Institute for Advanced Study, University of Chinese Academy of Sciences, Hangzhou, 310024 China

**Keywords:** Mesenchymal stem cells (MSCs), Single-cell RNA sequencing (scRNA-seq), Cell heterogeneity, Cytokine priming, Functional subpopulations

## Abstract

**Background:**

Mesenchymal stem cells (MSCs) hold immense promise for use in immunomodulation and regenerative medicine. However, their inherent heterogeneity makes it difficult to achieve optimal therapeutic outcomes for a specific clinical disease. Primed MSCs containing a certain cytokine can enhance their particular functions, thereby increasing their therapeutic potential for related diseases. Therefore, understanding the characteristic changes and underlying mechanisms of MSCs primed by various cytokines is highly important.

**Results:**

In this study, we aimed to reveal the cellular heterogeneity, functional subpopulations, and molecular mechanisms of MSCs primed with IFN-γ, TNF-α, IL-4, IL-6, IL-15, and IL-17 using single-cell RNA sequencing (scRNA-seq). Our results demonstrated that cytokine priming minimized the heterogeneity of the MSC transcriptome, while the expression of MSC surface markers exhibited only slight changes. Notably, compared to IL-6, IL-15, and IL-17; IFN-γ, TNF-α, and IL-4 priming, which stimulated a significantly greater number of differentially expressed genes (DEGs). Functional analysis, which included Gene Ontology (GO) and Kyoto Encyclopedia of Genes and Genomes (KEGG) analyses, indicated that IFN-γ, TNF-α, and IL-4-primed hUC-MSCs are involved in interferon-mediated immune-related processes, leukocyte migration, chemotaxis potential, and extracellular matrix and cell adhesion, respectively. Moreover, an investigation of various biological function scores demonstrated that IFN-γ-primed hUC-MSCs exhibit strong immunomodulatory ability, TNF-α-primed hUC-MSCs exhibit high chemotaxis potential, and IL-4-primed hUC-MSCs express elevated amounts of collagen. Finally, we observed that cytokine priming alters the distribution of functional subpopulations of MSCs, and these subpopulations exhibit various potential biological functions. Taken together, our study revealed the distinct regulatory effects of cytokine priming on MSC heterogeneity, biological function, and functional subpopulations at the single-cell level.

**Conclusions:**

These findings contribute to a comprehensive understanding of the inflammatory priming of MSCs, paving the way for their precise treatment in clinical applications.

**Supplementary Information:**

The online version contains supplementary material available at 10.1186/s13578-024-01219-3.

## Background

Mesenchymal stem cells (MSCs) hold great potential for the treatment of various clinical disease types, including bone and cartilage defects, cardiovascular disease, neurological degeneration, liver disorders, immunological diseases, graft versus host disease (GvHD), and Crohn’s disease [1, 2]. Several biological functions of MSCs participate in treating different diseases, including immunomodulation, homing to the injury site, and paracrine signalling [3, 4]. Studies have demonstrated that MSCs play an important role in tissue homeostasis and immunomodulation through interactions with immune cells and the secretion of factors, including growth factors, cytokines, and antifibrotics [5, 6]. Notably, their immunosuppressive capacity is primarily achieved by producing anti-inflammatory molecules, such as prostaglandin E2 (PGE-2) and TNFα-stimulated gene-6 (TSG-6), to inhibit NK cells and effector T cells.

MSCs are heterogeneous cells that possess diverse functions and multipotentialities. The cellular heterogeneity of MSCs is associated with several subpopulations focused on their proliferation, multipotency, and immunomodulatory capabilities [7, 8]. The heterogeneity of MSCs, which may originate from differences in tissue sources, culture methods, and expansion levels, may all influence therapeutic efficacy, hindering therapeutic efficacy and ultimately posing difficulties in clinical trials and blocking the development of MSC products [2]. Different clusters of MSCs may react differently to inflammatory priming, leading to inconsistent treatment efficacy. Therefore, identifying appropriate methods to reduce the heterogeneity of MSCs and promote their polarization to the same phenotype is necessary.

MSCs are highly plastic, and their phenotype and biological function depend on the state of their microenvironment, which includes the inflammatory microenvironment in immune-mediated diseases and the hypoxic microenvironment in diseases causing local tissue damage [9]. MSCs can sense dynamic inflammatory changes in the microenvironment and interact with the innate and adaptive immune systems to restore balance to the microenvironment [10]. MSCs can exert immunomodulatory effects through a direct pathway without prelicensing or reciprocal regulation that requires cytokine activation in vitro or in vivo, and their specific immunosuppressive behaviors depend on specific environmental signals. This prelicensing process is called “MSC priming” [11]. In MSCs, robust expression of chemokines and immunosuppressive factors, such as CCL5, CXCL9, indoleamine 2,3-dioxygenase (IDO), prostaglandin-endoperoxide synthase 2 (PTGS-2), and TSG-6, can be induced by inflammatory cytokines, leading to immunosuppression via a concerted mechanism [12]. It is crucial to study the distinct factors responsible for MSC priming to aid in the treatment of different diseases. Various inflammatory cytokines have been reported to prime MSCs, and their function and therapeutic efficacy have been investigated across different diseases [13–19]. However, our understanding of the disease microenvironment and its impact on MSC activation requires further development. Additionally, additional information is needed about the types of primed MSCs that are optimal for specific therapeutic actions.

Single-cell RNA sequencing (scRNA-seq) is a recent innovation that allows massively parallel analysis of gene expression profiles at the single-cell level. This approach has become a powerful tool for investigating tissue and cell heterogeneity and for comprehensively dissecting cellular heterogeneity in an unbiased manner without requiring prior information regarding the cell population [20, 21]. Therefore, understanding cellular heterogeneity under cytokine priming is effective. Lu et al. analysed the scRNA-seq data of human bone marrow-derived MSCs (hBM-MSCs) in the presence or absence of IFN-γ and TNF-α priming and reported the expression profiles of unprimed and primed hBM-MSCs related to the cell cycle, stemness, and immunomodulatory capability at single-cell resolution. This approach assisted in developing a comprehensive understanding of the inflammatory priming of hBM-MSCs and further clinical applications [22].

Numerous reports have established a connection between elevated levels of inflammatory cytokines and immune-mediated diseases, such as IL-1RA, IL-6, and IL-18 in COVID-19 patients [23]; TNF-α, IL-1, IL-6, IL-15, and IL-17 A in rheumatoid arthritis (RA) [24, 25]; TNF, IFN-γ, and IL-6 in systemic lupus erythematosus (SLE) [26]; and IL-4, IL-5 and IL-13 in the pathogenesis of asthma and other TH2 cell/IgE-mediated immune diseases [27]. Therefore, these cytokines are associated with the inflammatory microenvironment in many immune-mediated diseases and participate in disease pathogenesis and progression. In SLE, IFN-γ is produced by NK cells early in the immune response; however, once the adaptive immune system is activated, T cells are the major producers of this cytokine. IFN-γ is an important mediator of inflammation and immunity and has the capacity to induce and regulate several proinflammatory cytokines [26]. In RA, TNF-α, IL-1, and IL-17 are produced by Th17 cells. In addition, TNF-α induces monocyte activation and cytokine release and can reduce synovial fibroblast proliferation and collagen synthesis; additionally, IL-17 induces leukocyte cytokine production, synovial fibroblast cytokine production and MMP release. IL-6 and IL-15 are produced by monocytes, T cells and synovial fibroblasts. IL-6 induces B-cell proliferation and antibody production and increases T-cell proliferation, differentiation, and cytotoxicity; IL-15 induces T-cell chemokineosis and activation, B-cell differentiation, NK-cell activation and cytotoxicity, and synovial fibroblast activation [25]; and in TH2 cell-mediated immune responses, IL-4 and IL-13 share the same receptor, and both are able to activate the signal transducer and activator of transcription 6 (STAT6) signalling pathway. These cytokines have proinflammatory and profibrotic effects on lung diseases, such as asthma [28].

In this study, we profiled the single-cell transcriptomes of hUC-MSCs after priming with six cytokines, IFN-γ, TNF-α, IL-4, IL-6, IL-15, and IL-17, to comprehensively investigate the cellular heterogeneity and biological function of unprimed and primed human umbilical cord mesenchymal stem cells (hUC-MSCs). We analysed cellular heterogeneity, surface markers, GO classifications and functional cluster analysis, DEGs, differentiative potencies, chemotaxis, immunomodulatory capability, and collagenic synthesis at single-cell resolution. This work was performed to determine the inflammatory priming of hUC-MSCs, their diverse biological functions, and their potential value for clinical application.

## Results

### Cytokine priming alters the heterogeneity of hUC-MSCs

**Fig. 1:**
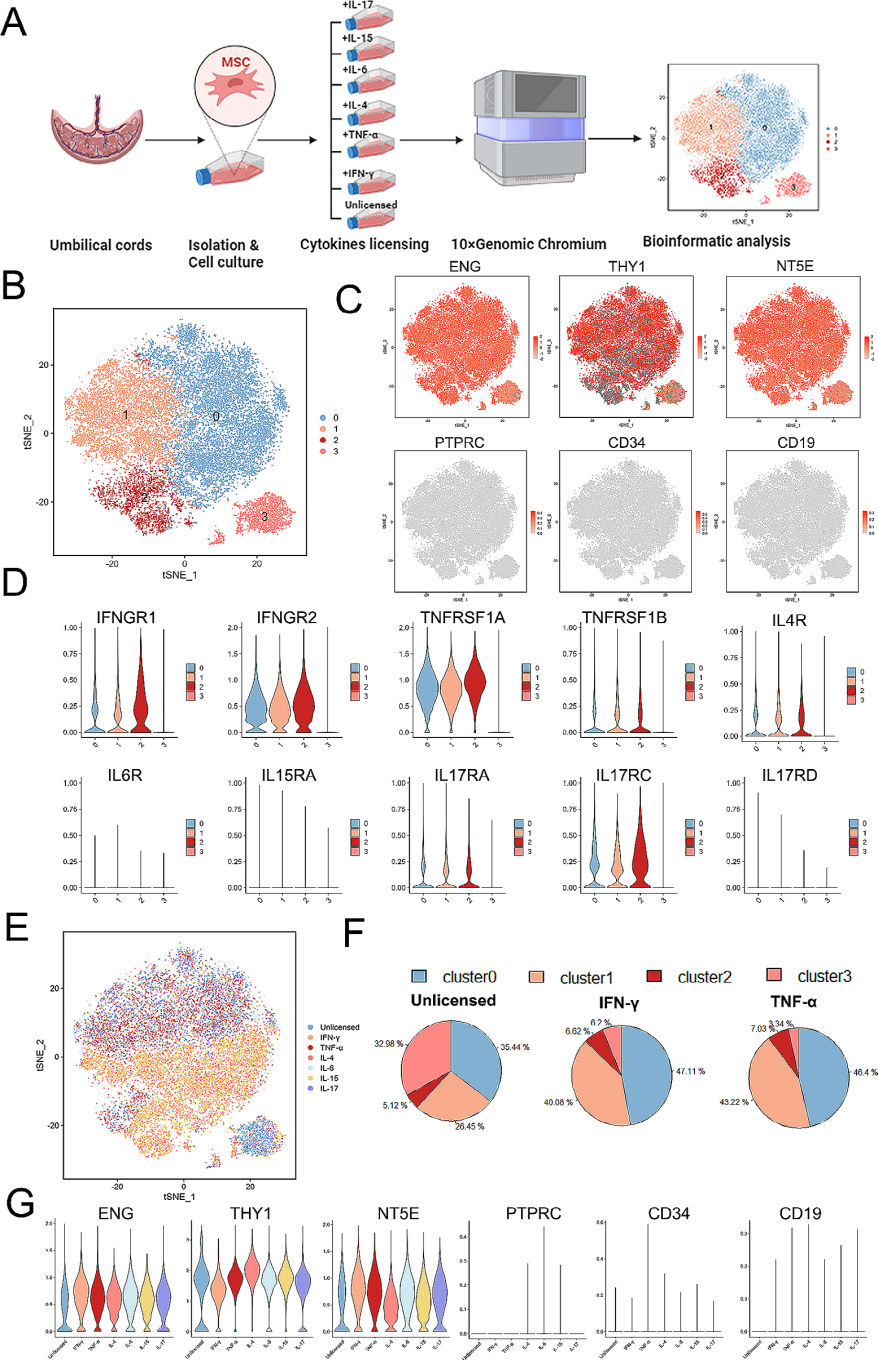
Cellular heterogeneity of hUC-MSCs before and after cytokine priming (**A**) Schematic overview of the study design. (**B**) t-SNE plots of the scRNA-seq clusters of hUC-MSCs. (**C**) t-SNE plots showing the expression levels of marker genes of hUC-MSCs. (**D**) Violin plots showing the expression levels of the receptor genes IFN-γ (IFNGR1, IFNGR2), TNF-α (TNFRSF1A, TNFRSF1B), IL-4 (IL4R), IL-6 (IL6R), IL-15 (IL15RA), and IL-17 (IL17RA, IL17RC, IL17RD). (**E**) t-SNE plots of the scRNA-seq data of hUC-MSCs after cytokine priming. (**F**) Sector graph illustrating the percentage change in the four clusters in unprimed, IFN-γ-primed, or TNF-α-primed hUC-MSCs; this change reflects the change in heterogeneity of the cell subpopulations. (**G**) Violin plots showing the expression levels of the marker genes of hUC-MSCs after cytokine priming

hUC-MSCs were isolated from UC Wharton’s jelly and cultured according to previously described methods [19]. These primary cells can be MSCs, including those that undergo tri-lineage differentiation into adipogenic, chondrogenic, and osteogenic cells (Additional file 1: Fig S1**A**). The hUC-MSCs were positive for typical mesenchymal cell surface markers (CD105, CD90, and CD73), while hematopoietic cell markers (CD45, CD34, and CD19) were almost entirely absent **(Additional file 1: Fig ****S1****B**). To analyse the biological characteristics of various cytokine-primed MSCs, hUC-MSCs were primed with IFN-γ, TNF-α, IL-4, IL-6, IL-15, and IL-17 *in vitro.* These samples were subsequently used to generate scRNA-seq data following the 10× Genomics protocol (Fig. 1A). After stringent cell filtration, we removed genes based on the following criteria: unique genes < 200 or > 9,000, number of unique molecular identifiers (UMI) < 2,000 or > 100,000, mitochondrial counts < 10% and ribosomal counts < 30%. A total of 23,250 cells were ultimately obtained for downstream analysis **(Additional file 1: Fig ****S1****C**). We isolated four cell clusters using a graph-based method alongside visualization via tSNE. The analysis demonstrated that hUC-MSCs had four distinct subpopulations: cluster 0, cluster 1, cluster 2 and cluster 3 (Fig. 1B). All the subpopulations in the tSNE plots were positive for the expression of ENG (CD105), THY1 (CD90), and NT5E (CD73) and negative for the expression of PTPRC (CD45), CD34, and CD19 (Fig. 1C). Multiple cytokines can potentially influence the function of MSCs in vivo following transplantation through ligand–receptor binding interactions. Subsequently, the expression of receptors for these six cytokines in MSC subpopulations was assessed through scRNA-seq. Our analysis revealed the expression of IFN-γ, TNF-α, IL-4, and IL-17 receptors in hUC-MSCs, while the receptors for IL-6 and IL-15 (IL6R and IL15RA) were expressed at low levels. Among these receptors, the IFN-γ receptor (IFNGR2) and TNF-α receptor (TNFRSF1A) exhibited greater expression than did the other receptors. Additionally, IFNGR1, TNFRSF1B, IL4R, IL17RA, and IL17RC exhibited moderate expression levels (Fig. 1D). Protein expression levels of surface receptors were assessed via flow cytometry analysis **(Additional file 1: Fig ****S1****D**). The results indicated that IFNGR1 and IL-17RA exhibited increased protein levels, whereas TNFR1, IL4R, IL6R, and IL15R demonstrated moderate expression. On the other hand, IFNGR2 and TNFR2 were found to be expressed at low levels **(Additional file 1: Fig ****S1****E**). Thus, it is likely that hUC-MSCs can respond to these cytokines. scRNA-seq further demonstrated that the priming of different cytokines could induce the differential distribution of MSC subpopulations. The distribution of some cytokine-primed hUC-MSCs was predominantly reduced, demonstrating that cytokines, especially IFN-γ and TNF-α, at the single-cell level could significantly lessen the transcriptomic heterogeneity of MSCs (Fig. 1E**).** In unprimed MSCs, three large subpopulations were identified (clusters 0, 1, and 3), and one small subpopulation was identified (cluster 2). Only two large clusters (clusters 0 and 1) persisted following IFN-γ and TNF-α priming, which demonstrated that cytokine priming could lower the heterogeneity of hUC-MSCs, especially when IFN-γ and TNF-α were used (Fig. 1F). While different cytokine-primed hUC-MSCs expressed MSC-related surface markers, the expression levels of these markers differed among the groups. For example, the expression of ENG and NT5E in IFN-γ-primed hUC-MSCs was slightly elevated compared to that in other groups, while their THY1 expression was the lowest (Fig. 1G). The heterogeneity and surface markers of hUC-MSCs can be altered by cytokine priming, but their biological function requires further analysis via scRNA-seq.

### Analysis of differentially expressed genes (DEGs) in hUC-MSCs primed with various cytokines

**Fig. 2:**
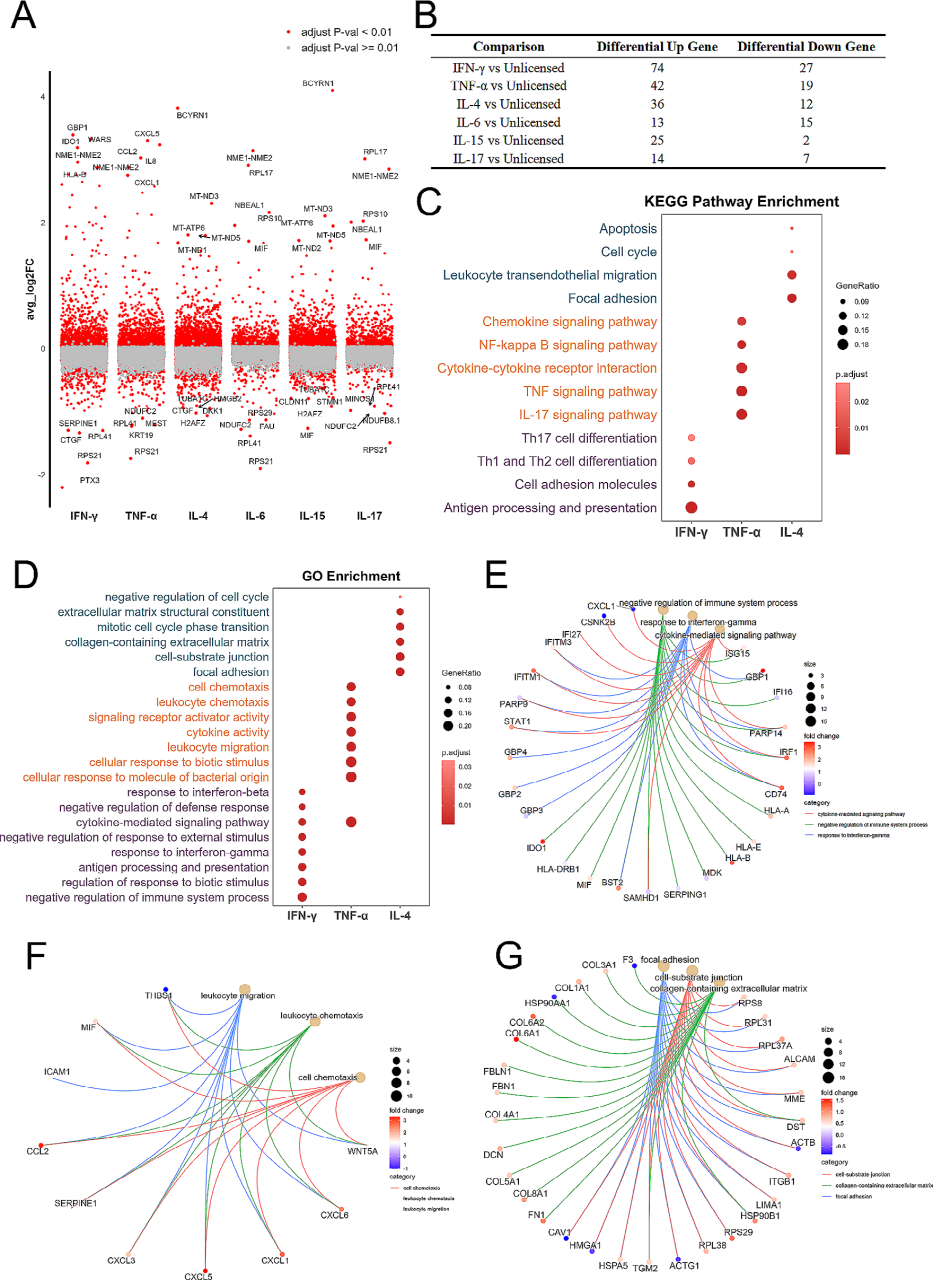
Predicted functions and pathways associated with DEGs after cytokine priming in hUC-MSCs. (**A**) Differential expression gene analysis showing upregulated and downregulated genes across cytokine-primed hUC-MSCs. An adjusted *p* value < 0.01 is indicated in red, while an adjusted *p* value > = 0.01 is indicated in gray. (**B**) Statistical table of genes significantly differentially expressed between cytokine-primed hUC-MSCs and unprimed hUC-MSCs. (C-D) KEGG (**C**) and GO (**D**) enrichment analyses of IFN-γ-, TNF-α-, or IL-4-primed hUC-MSCs; dot plot showing the most significant terms. The size of each dot indicates the gene ratio (the total number of DEG-enriched genes). The color indicates the adjusted *p* value for enrichment analysis. (**E**) GO enrichment network of IFN-γ-primed hUC-MSCs. (**F**) GO enrichment network of TNF-α-primed hUC-MSCs. (**G**) GO enrichment network of IL-4-primed hUC-MSCs

Compared to those in the unprimed hUC-MSCs, we found DEGs across IFN-γ, TNF-α, IL-4, IL-6, IL-15, and IL-17-primed hUC-MSCs **(Additional file 11–16: Table ****S2****-7**). GBP1, IDO1, WARS, HLA-B, and NME1-NME2 were highly expressed in IFN-γ-primed hUC-MSCs. CXCL5, CXCL1, CCL2, and IL8 were highly expressed in TNF-α-primed hUC-MSCs. Similarly, BCYRN1, MT-ND3, MT-ATP6, MT-ND5 and MT-ND1 were highly expressed in IL-4-primed hUC-MSCs. NME1-NME2, RPL17, NBEAL1, RPS10, and MIF were highly expressed in IL-6-primed hUC-MSCs. BCYRN1, MT-ND3, MT-ATP6, MT-ND5, and MT-ND2 were highly expressed in IL-15-primed hUC-MSCs. RPL17, and NME1-NME2, RPS10, NBEAL1, and MIF were highly expressed in IL-17-primed hUC-MSCs (Fig. 2A). To clarify the number of upregulated and downregulated DEGs across cytokine-primed hUC-MSCs and unprimed hUC-MSCs, we used the Seurat FindAllMarkers function for every sample. We performed a Wilcoxon rank sum test, with the DEGs of samples selected based on a *p* < 0.05 and a fold change > 0.8. The DEG data indicated that 101 DEGs (74 upregulated genes and 27 downregulated genes) were upregulated in IFN-γ-primed hUC-MSCs compared to unprimed hUC-MSCs; moreover, 61 DEGs (42 and 19), 48 DEGs (36 and 12), 28 DEGs (13 and 15), 27 DEGs (25 and 2), and 21 DEGs (14 and 7) were upregulated in the TNF-α, IL-4, IL-6, IL-15, and IL-17-primed hUC-MSC groups, respectively (Fig. 2B). There were fewer DEGs in the IL-6, IL-15, and IL-17-primed groups, and these three cytokines had minimal effects on cellular functions, as shown by GO and KEGG analyses. For example, ribosome function was enriched in IL-6- and IL-17-primed hUC-MSCs, and extracellular matrix (ECM) function was slightly altered in IL-15-primed hUC-MSCs **(Additional file 2: Fig. ****S2****A, B**). Considering the low expression of IL-6 and IL-15 receptors (IL6R and IL15RA) and moderate expression of IL-17 receptors (IL17RA and IL17RC) (Fig. 1D), we investigated these two groups of cells. Therefore, we investigated only IFN-γ-, TNF-α-, and IL-4-primed hUC-MSCs in the following analysis. Furthermore, Kyoto Encyclopedia of Genes and Genomes (KEGG) analysis demonstrated that antigen processing and presentation, cell adhesion, and T helper cell differentiation were enriched in IFN-γ-primed hUC-MSCs, whereas the tumor necrosis factor (TNF)/IL-17/NF-κB signalling pathway, chemokine signalling pathway, and cytokine‒cytokine receptor interaction were associated with TNF-α-primed hUC-MSCs, and focal adhesion and leukocyte transendothelial migration were enriched in IL-4-primed hUC-MSCs (Fig. 2C). Upon GO enrichment analysis, negative regulation of immune system processes (associated genes IDO1, GBP1, HLA-E, and CD74), response to interferon-gamma (associated genes GBP1-4, IFITM1, and STAT1), and cytokine-mediated signalling pathway (associated genes IFITM1, STAT1, IRF1, and PARP14), were associated with the IFN-γ-primed hUC-MSCs (Fig. 2D, E); leukocyte migration, leukocyte chemotaxis, and cell chemotaxis were enriched in TNF-α-primed hUC-MSCs (associated genes CXCL1, CXCL5, CXCL6, CCL2, and MIF) (Fig. 2D, F); adhesion pathways, such as focal adhesion and cell-substrate junction (associated genes RPS8, RPS29, RPL31, RPL38, ALCAM, MME, HSP90B1, and HSPA5), collagen-containing extracellular matrix (associated genes COL6A1, COL6A2, COL1A1, COL3A1, FN1, DCN, FBN1, and FBLN1), were associated with the IL-4-primed hUC-MSCs (Fig. 2D, G). Overall, we found that IFN-γ-primed hUC-MSCs can regulate the interferon-mediated immune system response, TNF-α-primed hUC-MSCs can promote leukocyte migration and chemotaxis, and IL-4-primed hUC-MSCs play key roles in focal adhesion, cell-substrate junctions, and the collagen-containing extracellular matrix.

### Gene set enrichment analysis of hUC-MSCs primed with various cytokines

**Fig. 3:**
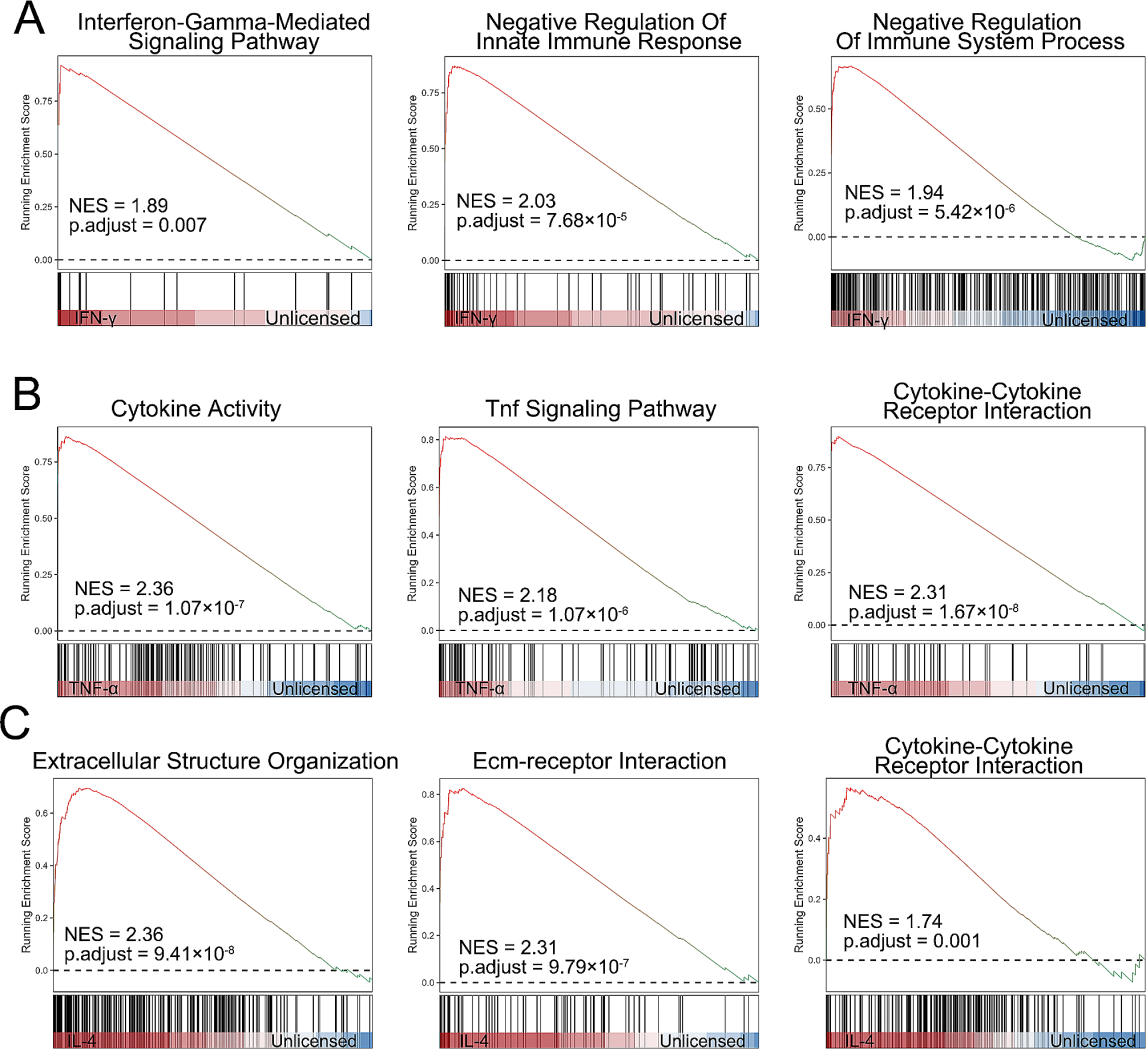
Gene set enrichment analysis of cytokine-primed hUC-MSCs. (**A**) Three representative significantly enriched gene sets from IFN-γ-primed hUC-MSCs; the normalized enrichment score and adjusted *p* value were calculated via permutation tests. (**B**) Three representative significantly enriched gene sets from TNF-α-primed hUC-MSCs; the normalized enrichment score and adjusted *p* value were calculated via permutation tests. (**C**) Three representative significantly enriched gene sets from IL-4-primed hUC-MSCs; the normalized enrichment score and adjusted *p* value were calculated via permutation tests

We performed GSEA on our scRNA-seq data using gene sets from the GO and KEGG databases to better understand the underlying mechanism of IFN-γ, TNF-α, or IL-4-primed hUC-MSCs and unprimed hUC-MSCs. As expected, compared with unprimed hUC-MSCs, IFN-γ-primed hUC-MSCs had increased interferon-gamma-mediated signalling pathway activity (NES = 1.89), increased negative regulation of the innate immune response (NES = 2.03), and increased negative regulation of immune system processes (NES = 1.94) (Fig. 3A). We also found that TNF-α-primed hUC-MSCs activated cytokine activity (NES = 2.36), the TNF signalling pathway (NES = 2.18), and cytokine‒cytokine receptor interactions (NES = 2.31) (Fig. 3B). Similarly, our GSEA showed that IL-4-primed hUC-MSCs had an activated extracellular structure organization (NES = 2.36), an enhanced ECM-receptor interaction (NES = 2.31), and a cytokine‒cytokine receptor interaction (NES = 1.74) (Fig. 3C). These results suggest that diverse cytokines stimulate various signalling pathways in IFN-γ-, TNF-α-, and IL-4-primed hUC-MSCs, possibly altering the biological functions of hUC-MSCs in vitro and in vivo.

### Analysis of chemotaxis, immunomodulation, and collagen synthesis

**Fig. 4:**
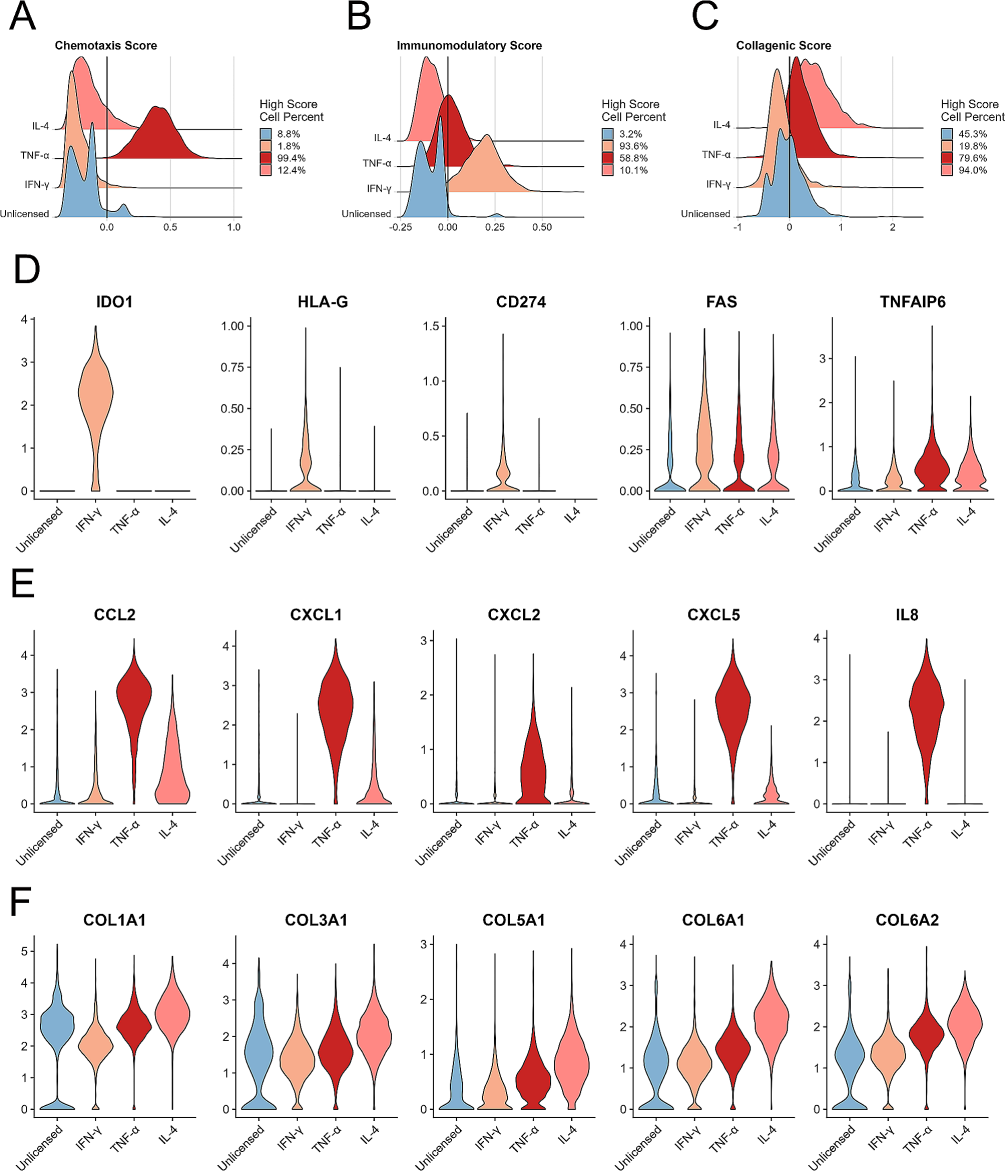
Cytokine-primed and unprimed hUC-MSCs exhibited different predictive efficacies. (**A**-**C**) Ridge plot showing the chemotaxis score (**A**), immunomodulation score (**B**), and collagenic score (**C**) of hUC-MSCs with or without cytokine priming. The zero line was established as a threshold for discriminating cell potential, and the percentage of high-score cells is also shown. (**D**) Violin plots showing immunomodulatory-related gene expression after cytokine priming. (**E**) Violin plots showing chemotaxis-related gene expression after cytokine priming. (**F**) Violin plots showing collagen-related gene expression after cytokine priming. The Wilcoxon rank sum test was performed for significance; ****P < 0.0001

Tri-lineage differentiation gene score analysis suggested that there was no significant difference between unprimed and cytokine-primed hUC-MSCs, demonstrating that IFN-γ, TNF-α, or IL-4 priming had little effect on MSC differentiation, including adipogenic, chondrogenic, and osteogenic ability (Additional file 2: Fig S2C-E). Moreover, the chemotaxis gene score indicated that 99.4% of the TNF-α-primed hUC-MSCs had high chemotactic ability, while the other three groups had chemotactic ability, with 8.8% (unprimed), 1.8% (IFN-γ), and 12.4% (IL-4) (Fig. 4A). The immunomodulatory gene score indicated that 93.6% of the IFN-γ-primed hUC-MSCs and 58.8% of the TNF-α-primed hUC-MSCs had high immunomodulatory ability, while 3.2% of the unprimed hUC-MSCs and 10.1% of the IL-4-primed hUC-MSCs (Fig. 4B). The collagenic gene score showed that 94.0% of the IL-4-primed hUC-MSCs and 79.6% of the TNF-α-primed hUC-MSCs had high collagenic ability, whereas 45.3% of the unprimed hUC-MSCs and 19.8% of the IFN-γ-primed hUC-MSCs (Fig. 4C). We also assessed the consistency of the AddModuleScore results mentioned above by utilizing methods based on the gene expression ranking of a single sample, such as AUCell and Ucell. The results obtained from the AUCell and Ucell methods were consistent with the results obtained from the AddModuleScore (Additional file 3: Fig S3A-C). To confirm the accuracy of these scores, we performed functional experiments to enhance the applicability of our bioinformatics data. Specifically, we found that TNF-α-primed hUC-MSCs exhibited greater chemotactic migration than did the other groups (Additional file 4: Fig S4A, B). Additionally, compared with those in the other MSC groups, the immunosuppressive ability of IFN-γ-primed hUC-MSCs was greater (Additional file 4: Fig S4C). Moreover, the collagen secretion assay results indicated significant increases in the collagen I and collagen V levels in comparison to those in the other three groups (Additional file 4: Fig S4D-F). These functional findings provide further confirmation and validation of the scRNA-seq results described in Fig. 4A-C. scRNA-seq data also revealed that IFN-γ-primed hUC-MSCs expressed high levels of immunomodulatory genes, including IDO1, HLA-G, CD274, and FAS (*p* ≤ 0.0001; Fig. 4D). The expression of chemotactic-associated genes, including CCL2, CXCL1, CXCL2, CXCL5, and IL8 (CXCL8), was significantly upregulated in TNF-α-primed hUC-MSCs compared to unprimed hUC-MSCs (*p* ≤ 0.0001; Fig. 4E). The expression of the collagen-associated genes COL1A1, COL3A1, COL6A1, COL6A2, and COL5A1 was greater in the IL-4-primed hUC-MSCs than in the other three groups (*p* ≤ 0.0001; Fig. 4F). The duration of cytokine priming may influence the expression of functional genes in hUC-MSCs. We selected five time points for cytokine priming with IFN-γ, TNF-α, and IL-4: 6 h, 12 h, 24 h, 36 h, and 48 h. The results of our qPCR analysis indicated that the expression of immunomodulatory genes, chemotactic genes, and collagen genes in hUC-MSCs was significantly influenced by the duration of cytokine priming. Specifically, the optimal priming times were identified as 24 or 36 h, as these time points had the most pronounced effects on gene expression (Additional file 5: Fig S5A-C). In the present study, only single donor-derived hUC-MSCs were primed with various cytokines, and single-cell bioinformatics data were analysed. Considering the cellular heterogeneity among different individuals, we included qPCR data from 3 different donors (Additional file 6: Fig S6A-C). The results indicated that the expression levels of immunomodulatory genes (IDO1 and PDL1), chemotaxis genes (IL-8 and CXCL1), and collagen genes (COL3A1 and COL8A1) were different among these three donors. However, many genes exhibit increased expression in these three donors following IFN-γ, TNF-α and IL-4 priming. This trend was similar to the findings from the scRNA-seq data shown in Fig. 4D-F. Together, the scRNA-seq data demonstrated that IFN-γ-primed hUC-MSCs possess a strong immunomodulatory ability, TNF-α-primed hUC-MSCs exhibit high chemotaxis, and IL-4-primed hUC-MSCs express elevated levels of collagens.

### Changes in the subpopulations of hUC-MSCs primed by various cytokines

**Fig. 5:**
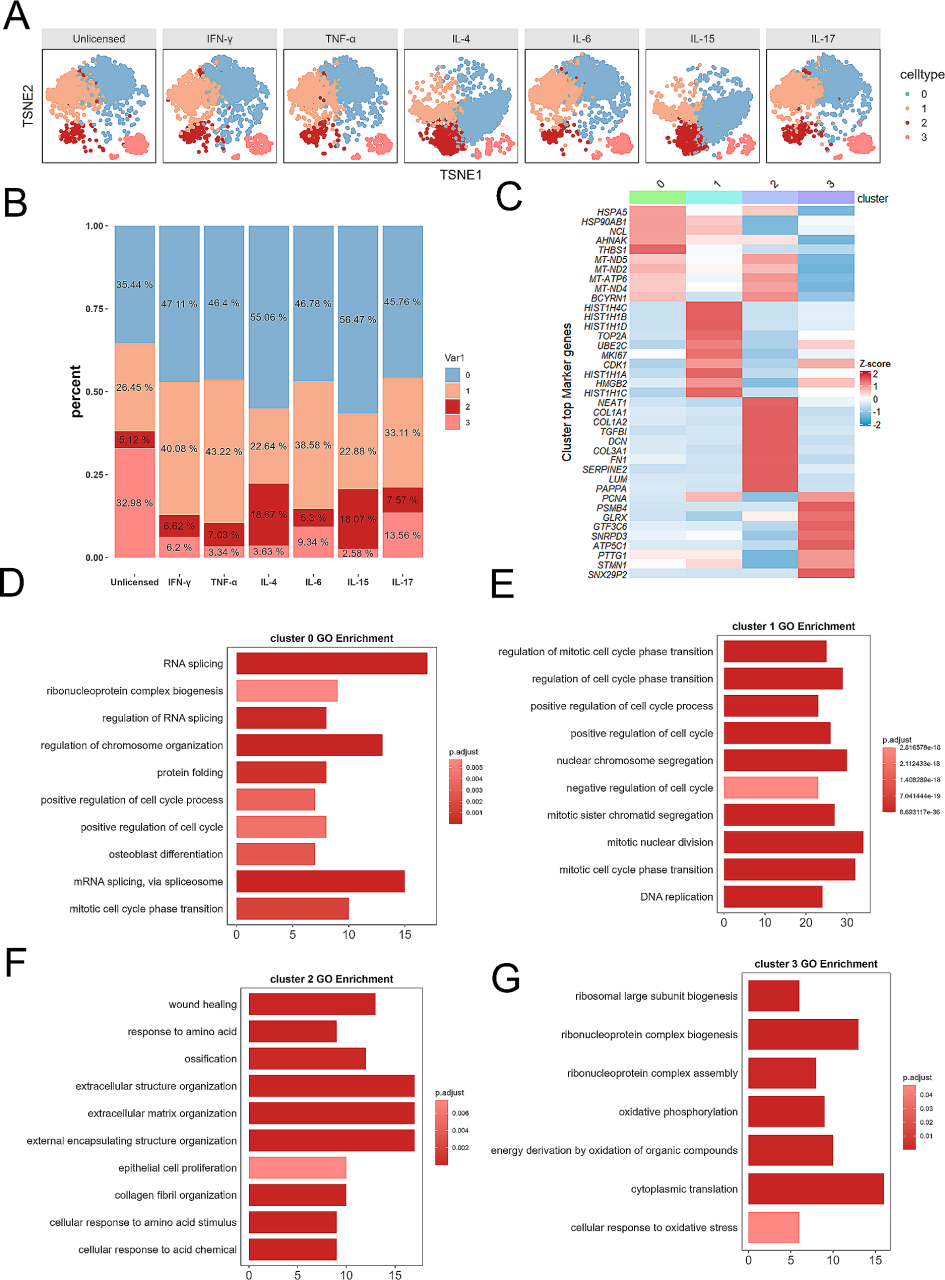
Single-cell RNA sequencing analysis revealed functional heterogeneity among the different clusters. (**A**) Cell type identification in the t-SNE plot of cytokine-primed and unprimed hUC-MSCs. (**B**) The relative contribution of each cluster was weighed using the number of cells per sample and scaled to 100%. (**C**) Heatmap of the top 10 DEGs in each cluster. (**D**) GO enrichment analyses of cluster 0. (**E**) GO enrichment analyses of cluster (1) (**F**) GO enrichment analyses of cluster (2) (**G**) GO enrichment analyses of cluster 3

Using a graph-based method and visualization through t-distributed stochastic neighbor embedding (tSNE), we found four cell clusters in unprimed hUC-MSCs. Many cells were distributed in cluster 0 and cluster 1, demonstrating that these two cell groups are the main subpopulations of hUC-MSCs, while cluster 2 and cluster 3 were relatively smaller. We also found that, compared with no priming, priming with different cytokines could alter the distribution of MSC subpopulations; for example, cluster 2 in IL-4-primed and IL-15-primed hUC-MSCs (Fig. 5A). Due to the change in cell distribution, different MSC subpopulations were upregulated or downregulated after cytokine priming. Compared to those of unprimed hUC-MSCs, the percentage of cells in cluster 0 was greater after IFN-γ, TNF-α, IL-4, IL-6, IL-15, and IL-17 priming, especially for IL-4-primed and IL-15-primed hUC-MSCs. Cluster 1 included IFN-γ-, TNF-α-, IL-6-, and IL-17-primed hUC-MSCs. The expression of cluster 2 genes was increased largely in IL-4- and IL-15-primed hUC-MSCs. The percentage of cells in cluster 3 was reduced mainly in the cytokine-primed hUC-MSCs compared to the unprimed hUC-MSCs (Fig. 5B). Subpopulation markers were identified, and the top ten DEGs are listed. Notably, cluster 2 expressed high levels of collagen fibril organization genes (COL1A1, COL1A2, and LUM) and wound healing genes (FN1 and SERPINE2) (Fig. 5C). GO enrichment analysis revealed cellular functions associated with clusters 0, 1, 2, and 3 (Fig. 5D-G). RNA/mRNA splicing, regulation of chromosome organization, and regulation of cell cycle processes were enriched in cluster 0 (Fig. 5D). Regulation of the cell cycle process, mitotic nuclear division, and DNA replication were enriched in cluster 1 (Fig. 5E). Extracellular structure organization, extracellular matrix organization, external encapsulating structure organization, and wound healing were enriched in cluster 2 (Fig. 5F). Cytoplasmic translation, ribonucleoprotein complex biogenesis/assembly, and oxidative phosphorylation/stress were enriched in cluster 3 (Fig. 5G).

### Changes in various biological functions of the hUC-MSC subpopulations after cytokine priming

**Fig. 6:**
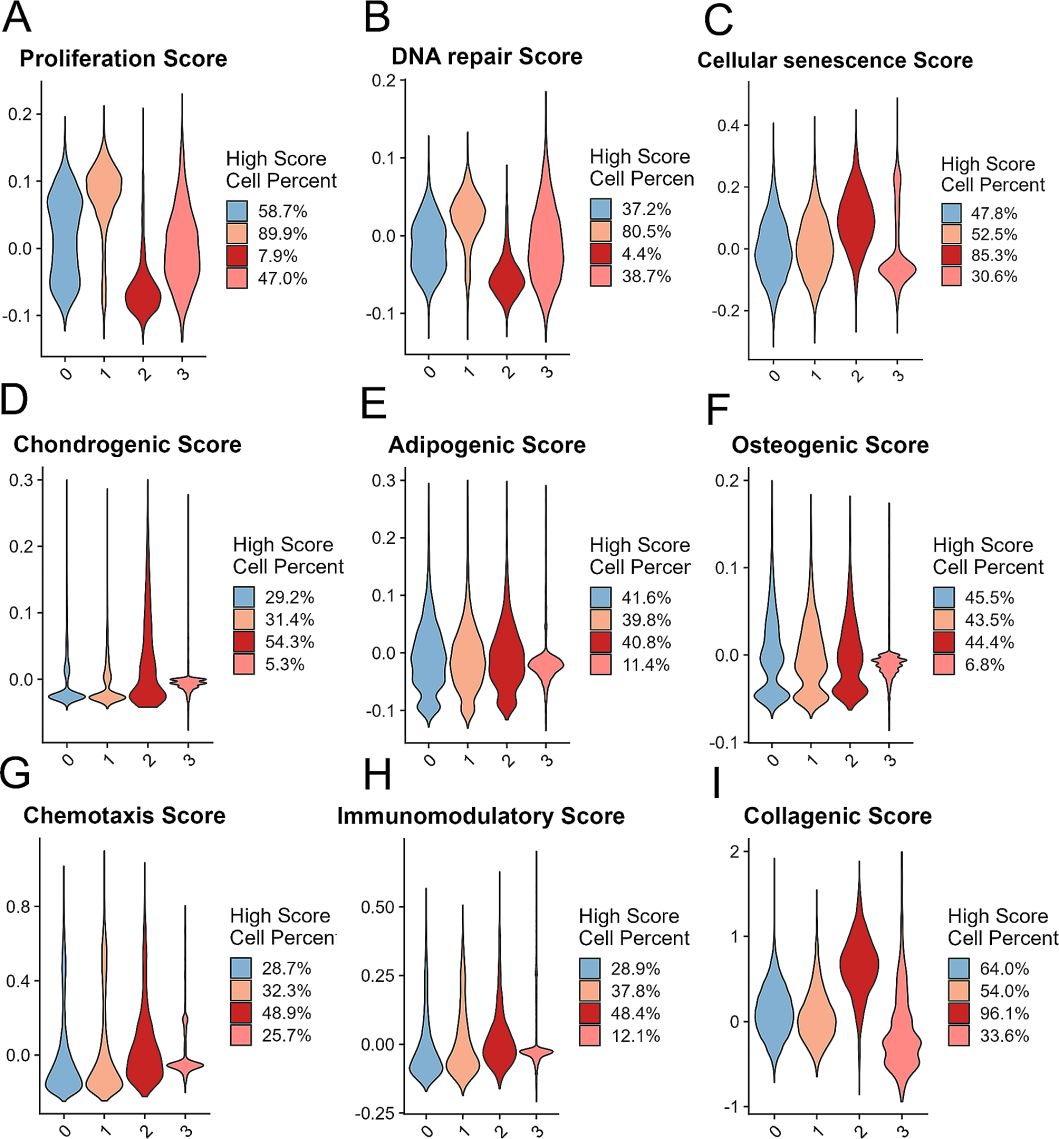
Different clusters had different predicted biological potencies. (A-F) Violin plots showing the proliferation score (**A**), DNA repair score (**B**), cellular senescence score (**C**), chondrogenic score (**D**), adipogenic score (**E**), osteogenic score (**F**), chemotaxis score (**G**), immunomodulatory score (**H**), and collagenic score (**I**) for the four candidate clusters

In consideration of regulation of cell cycle processes, DNA replication, and epithelial cell proliferation were enriched in the MSC clusters (Fig. 5D-G), we further analysed the proliferation, DNA repair, and cellular senescence scores in the different clusters. The results revealed that cluster 1 displayed an increased score for proliferation and DNA repair (Fig. 6A, B). Correspondingly, cluster 2 exhibited lower proliferation and DNA repair scores but a relatively greater cellular senescence score (Fig. 6C). Tri-lineage differentiation gene score analysis indicated that the hUC-MSCs in cluster 2 had greater chondrogenic potential than did those in cluster 0, cluster 1, and cluster 3 (54.3% vs. 29.2% vs. 31.4% vs. 5.3%). This difference may be related to the greater expression of extracellular matrix-associated genes in cluster 2 (Fig. 6D). In contrast, the adipogenic and osteogenic potentials were similar across Cluster 0, Cluster 1, and Cluster 2 (Fig. 6E, F). We found that, according to the chemotaxis gene score, 48.9% of the cells in cluster 2 exhibited high chemotactic ability, while 28.7% (cluster 0), 32.3% (cluster 1), and 25.7% (cluster 3) of the cells in the other subpopulations exhibited chemotactic ability (Fig. 6G). Cluster 2 had a higher immunomodulatory gene score than did the other subsets, with scores of 28.9% (cluster 0), 37.8% (cluster 1), 48.4% (cluster 2), and 12.1% (cluster 3) (Fig. 6H). Cluster 2 also demonstrated a significantly greater collagenic score than did the other subsets, with scores of 64.0% (cluster 0), 54.0% (cluster 1), 96.1% (cluster 2), and 33.6% (cluster 3) (Fig. 6I). Additionally, various cellular biological functions, including proliferation and senescence, tri-lineage differentiation, chemotaxis, immunomodulation, and collagen synthesis, were further evaluated in different clusters following priming with the six cytokines. (Additional file 7–9: Fig S7-9). First, IL-4- and IL-15-primed hUC-MSCs had significantly lower proliferation and DNA repair scores than other hUC-MSCs did, especially in cluster 0, cluster 2, and cluster 3 (*p* ≤ 0.0001; Additional file 7: Fig S7A, B), while they had relatively higher cellular senescence scores in cluster 0, cluster 1, and cluster 2 (*p* ≤ 0.001; Additional file 7: Fig S7C). Moreover, compared to those of other cytokine-primed hUC-MSCs, the chondrogenic score of IL-4- and IL-15-primed hUC-MSCs was greater in cluster 2 (*p* ≤ 0.0001; Additional file 8: Fig S8A), while in cluster 3, IL-4-hUC-MSCs exhibited strong chondrogenic, adipogenic, and osteogenic potential (*p* ≤ 0.001; Additional file 8: Fig S8A-C). More importantly, compared to those of other cytokine-primed hUC-MSCs, our findings demonstrated that cluster 0, cluster 1, cluster 2, and cluster 3 all exhibited greater chemotaxis in TNF-α-primed hUC-MSCs (*p* ≤ 0.0001); moreover, compared with unprimed hUC-MSCs, IL-17-primed hUC-MSCs exhibited an increased chemotaxis score (Additional file 9: Fig S9A). The results demonstrated that the IFN-γ-primed hUC-MSCs exhibited greater immunomodulatory potential than the other groups in all four clusters (*p* ≤ 0.0001); additionally, we observed an increased immunomodulatory score in cluster 0, cluster 1, and cluster 2 of the TNF-α-primed hUC-MSCs compared to the unprimed hUC-MSCs (Additional file 9: Fig S9B). Furthermore, IL-4-primed hUC-MSCs exhibited a relatively greater degree of collagen synthesis than did the other groups in cluster 0, cluster 1, and cluster 2 (*p* ≤ 0.001; Additional file 9: Fig S9C). Together, the different subpopulations of hUC-MSCs had different biological functions, and compared with those of cluster 0, cluster 1 and cluster 3, cluster 2 exhibited strong potential for chondrogenic ability, chemotaxis capacity, immunomodulatory potential, and collagen secretion; however, cluster 2 demonstrated less potential for cell proliferation than the other clusters.

## Discussion

MSCs from umbilical cord tissue are an ideal cell source for clinical applications with numerous advantages, including easy accessibility and availability in large quantities, high proliferation and differentiation potential, and low immunogenicity [29, 30]. Previous studies have demonstrated that cultured hUC-MSCs are heterogeneous populations containing variable subsets [31]. scRNA-seq is a powerful tool for exploring the heterogeneity and functionality of MSC subpopulations. By sequencing individual cells, researchers can identify distinct subgroups within MSC populations and gain insight into their gene expression profiles, signalling pathways, and differentiation potential [32]. Additionally, single-cell analysis may reveal rare or low-abundance cell types that may be missed using traditional bulk sequencing methods. This technology could improve our understanding of MSC biology and advance the development of MSC-based therapies for various diseases. In this study, we applied scRNA-seq to explore the effects of multiple cytokines (IFN-γ, TNF-α, IL-4, IL-6, IL-15, and IL-17) on the heterogeneity and biological function of hUC-MSCs.

Single-cell sequencing studies have revealed heterogeneity in the surface marker expression profiles of different MSC subpopulations [33]. Our scRNA-seq data revealed four different cell subtypes of bulk hUC-MSCs, with some (clusters 0 and 1) expressing high levels of several markers, such as CD73 (NT5E), CD90 (THY1), and CD105 (ENG), while clusters 2 and 3 had relatively lower expression of CD90. Xie et al. identified three clusters of bone marrow-derived MSCs through scRNA sequencing and subsequent bioinformatic analysis [34]. While their MSCs were positive for CD29, CD44, and CD105 and negative for CD14, CD45, and HLA-DR, these 3 clusters had different characteristics. The first cluster was determined to be the stemness subpopulation, the second cluster was identified as the functional subpopulation, and the third cluster was defined as the proliferative subpopulation. In this study, our 4 clusters may also have different features and diverse responses to inflammatory priming, which may impede standard clinical application, and a comprehensive understanding of the responses of heterogeneous MSCs to inflammatory priming is needed. We found that treatment of hUC-MSCs with specific cytokines reduces cellular heterogeneity, as evidenced by changes in the distribution of different subpopulations. For example, cell subgroups may be more clustered or lack a certain cell subgroup. Consistent with this phenomenon, Szabó et al. reported that IFN-γ and TNF-α preconditioning synchronized murine MSCs and attenuated donor-imprinted functional heterogeneity. They also found that priming with inflammatory cytokines abolishes the heterogeneity of immunosuppressive functions in MSC populations [35]. However, distinct cytokine priming cannot completely alter the expression of MSC markers but rather slightly increases or decreases their expression. For example, in our study, compared with unprimed hUC-MSCs, IFN-γ-primed hUC-MSCs exhibited increased expression of CD105 but reduced expression of CD90, upregulated expression of CD90, and downregulated expression of CD73 in IL-4-primed hUC-MSCs. Some non-MSC-related surface markers, including CD45, CD34, and CD19, are not constitutively expressed before or after priming.

The number of differentially expressed genes (DEGs) in hUC-MSCs treated with different cytokines also varied greatly. Our statistical data showed that 101, 61, and 48 DEGs were altered significantly in the IFN-γ, TNF-α, and IL-4-primed hUC-MSCs, respectively, which was far greater than that in the IL-6 (28 DEGs), IL-15 (27 DEGs), and IL-17 (21 DEGs) groups. In addition, the GO and KEGG analyses of these DEGs assisted us in predicting the specific biological functions of the different primed hUC-MSCs. Many DEGs related to IFN-γ-primed hUC-MSCs were enriched in antigen processing and presentation, cell adhesion molecules, and Th1/Th2/Th17 cell differentiation. In contrast, the TNF signalling pathway, cytokine‒cytokine receptor interaction pathway, and chemokine signalling pathway were associated with TNF-α-primed hUC-MSCs, while focal adhesion and leukocyte transendothelial migration were related to IL-4-primed hUC-MSCs. Ribosome-related functions were enriched in the IL-6– and IL-17-primed hUC-MSCs, and extracellular matrix/structure organization factors were enriched in the IL-15-primed hUC-MSCs. KEGG data revealed that the regulation of immune system processes, response to interferon-gamma, and cytokine-mediated signalling pathways were associated with IFN-γ-primed hUC-MSCs. Leukocyte migration and leukocyte/cell chemotaxis were associated with TNF-α-primed hUC-MSCs. Focal adhesion, cell-substrate junctions, and the collagen-containing extracellular matrix were associated with IL-4-primed hUC-MSCs. Through GO and KEGG analyses, we predicted that IFN-γ-primed hUC-MSCs are related to the immune regulation process and that TNF-α-primed hUC-MSCs are associated with the chemokine signalling pathway and leukocyte migration, whereas IL-4-primed hUC-MSCs are enriched in cell adhesion and the collagen-related extracellular matrix. Klinker et al. demonstrated that IFN-γ enhances the immunosuppressive capacity of all MSC lines, and morphology was used to predict the magnitude of IFN-γ-enhanced immunosuppressive activity [36]. Moreover, preconditioning MSCs with the inflammatory cytokine IFN-γ enhances not only their immunosuppressive activity but also their expression of HLA genes. An increase in the level of HLA-DR on IFN-γ-primed MSCs can elicit transplant rejection after cell transplantation, which can lead to increased MSC death and the induction of allogeneic immune reactions [37, 38]. Methods for reducing the rejection of allogeneic IFN-γ-primed MSCs may be necessary for overcoming the limitations of MSCs in clinical translation [39]. No reports associated with the biological functions of IL-4 in MSCs have been published, and our study is the first to uncover the functions of IL-4. Based on the differences in gene expression among unprimed hUC-MSCs, IFN-γ-primed hUC-MSCs, TNF-α-primed hUC-MSCs, and IL-4-primed hUC-MSCs, we compared several MSC-related biological function scores of these cells, including the adipogenic score, chondrogenic score, osteogenic score, chemotaxis score, immunomodulatory score, and collagenic score. Our results demonstrated that cytokine priming did not change the differential potential of TNF-α, but TNF-α priming enhanced chemotaxis, immunomodulation, and collagenic scores. Moreover, TNF-α plays the most important role in chemotaxis and can promote the expression of various chemokines, such as CCL2, CXCL1, 2, 5, and 8. IFN-γ priming primarily increases the immunomodulatory score and enhances the expression of IDO1 and HLA-G. IL-4 priming has a major role in increasing the collagenic score and largely increases the expression of COL1A1, COL3A1, COL6A2, and COL5A1. Consistent with our results, Lu et al. reported that IFN-γ + TNF-α-primed MSCs expressed high levels of CCL2, 5, 8, CXCL9, 10, and 11, and the expression of IDO1, PTGS2 (COX2), and IL-6 was upregulated in primed MSCs compared to unprimed MSCs [22]. Overall, through DEG analysis, we determined that different cytokines have diverse effects on hUC-MSCs and can enhance some of their biological characteristics, including chemotaxis, immunomodulation, and collagen secretion.

Recent evidence suggests that MSCs are mixed-cell populations composed of different cell subsets with different biological functions [40–42]. In our scRNA-seq study, to identify potential MSC subsets, we identified four clusters of hUC-MSCs. Different priming agents (IFN-γ, TNF-α, IL-4, IL-6, IL-15, and IL-17), especially IL-4 and IL-15, can change the cellular distribution of cytokines, as shown in the tSNE plot. The cell ratios of clusters 0, 1, 2, and 3 were increased or decreased after cytokine priming compared to those of unprimed hUC-MSCs. Cluster 0 was increased in every cytokine-primed group, cluster 3 was downregulated in every group, and cluster 2 was predominantly upregulated in IL-4- and IL-15-primed hUC-MSCs. Moreover, we also predicted the GO functional enrichment of clusters 0, 1, and 2. Cluster 0 included RNA/mRNA splicing, regulation of chromosome organization, and regulation of cell cycle-related functions. Cluster 1 included mitotic and cell cycle-related functions. Cluster 2 has major roles in extracellular matrix/structure organization and collagen organization. Cluster 3 included proteins involved in ribonucleoprotein complex biogenesis and energy derivation by the oxidation of organic compounds. We found that cluster 1 displayed an increased score for proliferation and DNA repair, and cluster 1 expressed proliferation-related genes such as TOP2A, UBE2C, MKI67, and CDK1. Based on these findings, cluster 1 can be considered a “proliferative MSC” phenotype [43]. Additionally, we found that clusters 0, 1, 2, and 3 all exhibited chondrogenic/adipogenic/osteogenic differentiation, chemotaxis ability, immunomodulatory potential, and collagenic secretion. Among these 4 clusters, cluster 2 had higher scores for chondrogenic differentiation, chemotaxis ability, immunomodulatory potential, and collagenic secretion. In contrast, cluster 2 exhibited lower scores for proliferation and DNA repair functions than did the other clusters, and cluster 2 expressed niche-supporting genes, including LUM, DCN, FN1, COL1A1, and COL3A1. Therefore, cluster 2 can be considered a “niche-supporting MSC” phenotype [43]. Therefore, we predict that various clusters of cytokine-primed hUC-MSCs exhibit distinct biological functions, emphasizing the importance of selecting the most suitable subpopulation for clinical application.

## Conclusions

Our work illustrates the heterogeneity of unprimed hUC-MSCs and the expression profiles of IFN-γ, TNF-α, IL-4, IL-6, IL-15, and IL-17-primed hUC-MSCs, as well as their subpopulations, as related to their immunomodulatory capability, chemotaxis ability, cell‒cell adhesion, and collagen-related extracellular matrix at single-cell resolution. This study may contribute to a comprehensive understanding of the inflammatory priming of hUC-MSCs and their further clinical application.

## Materials and methods

### Cell isolation and culture

Human umbilical cord mesenchymal stem cells were isolated and cultured as described below [44]. First, the umbilical cord was washed twice with 75% alcohol, followed by an additional two washes with Dulbecco’s phosphate-buffered saline (D-PBS); Invitrogen). After removing the arteries and veins, the mesenchymal tissue was dissected into approximately 1–2 mm pieces. The tissue clumps were added to T75 flasks supplemented with Dulbecco’s modified Eagle’s medium (DMEM, HyClone) supplemented with 5% (v/v) hPL (UltraGRO^TM^-Advanced, GMP Grade, AventaCell BioMedical), 2 mM L-glutamine, and 1% penicillin/streptomycin. After 12 days of culture, all the tissue clumps were removed, and the cells were cultured for an additional week. All the hUC-MSCs were maintained at 37 °C in a 5% CO_2_ incubator, and the medium was changed every 2–3 days. When the cells reached 80–90% confluence, they were passaged and cultured under the conditions mentioned above. P4 hUC-MSCs were used in this study.

### Cytokine priming in vitro

hUC-MSCs were seeded in 25cm^2^ flasks at a density of 1 × 10^4^ cells/cm^2^ in 3 mL DMEM (HyClone) complete medium, supplemented with 5% (v/v) hPL, 2 mM L-glutamine, and 1% penicillin/streptomycin. When nearly all the cells had adhered and exhibited normal fibroblastic morphology, at approximately 80% confluence, purified recombinant human cytokines (R&D System Minneapolis, MN, USA) were added separately to DMEM complete medium at the following concentrations for each cytokine: IFN-γ (25 ng/mL), TNF-α (10 ng/mL), IL-4 (50 ng/mL), IL-6 (100 ng/mL), IL-15 (100 ng/mL), and IL-17 (100 ng/mL). Following incubation for 24 h in complete medium, the cells were harvested using trypsin and evaluated for gene expression profiling. hUC-MSCscon served as the control group without prelicensing.

### Lineage differentiation in vitro

For osteogenic, adipogenic, and chondrogenic differentiation of hUC-MSCs in vitro, hUC-MSCs were cultured in the relevant differentiation media for 2 to 3 weeks following the corresponding protocols and analysed via Alizarin Red, Oil Red O, and toluidine blue staining, respectively. All the experimental procedures were performed according to the manufacturer’s manuals (Cyagen Biosciences, China).

### Flow cytometry sorting and analysis

Cytometric evaluation of the characteristic marker genes of hUC-MSCs was carried out beginning with treatment with trypsin and washing twice with PBS at P4. Flow cytometry was used to sort the hUC-MSCs and detect specific surface markers (positive for CD90, CD73, and CD105 and negative for CD45, CD34, and CD19). All the antibodies used were purchased from BD Pharmingen (San Diego, CA, United States). Antibodies against eight cytokine-related receptors were purchased from Biolegend (San Diego, CA, United States). At least 10,000 events were obtained using a BD™ Aria IIu flow cytometer (BD Bioscience), and the data were analysed using FlowJo 7.5 software (Treestar, Ashland, OR, United States).

### Single-cell library preparation

Following the manufacturer’s recommendations, single-cell RNA sequencing (scRNA-seq) libraries were prepared using the 10x Genomics Chromium Platform. After the library was constructed and cDNA was amplified, the library quality was evaluated using a Qubit (Thermo Fisher Scientific) and Agilent 2100 Bioanalyzer (Agilent Technologies, California). The barcoded libraries were sequenced using an Illumina NovaSeq 6000 with a sequencing depth of at least 100,000 reads per cell in 150 bp paired-end (PE150) mode.

### Preprocessing of scRNA-seq data

The raw sequencing data were subjected to base calling, adaptor trimming, and demultiplexing using Cell Ranger (version 3.1.0) [45]. We used the R package (version 4.1.3) and Seurat (version 4.0.0) [46] to conduct quality control, clustering, and differentiation analyses. Cells with fewer than 300 genes were removed, and genes that were not expressed in any cells were excluded based on the following criteria: unique genes < 200 or > 9,000, mitochondrial counts < 10%, ribosomal counts < 30%, and number of unique molecular identifiers (UMI) < 2,000 or > 100,000.

### Integrated analysis of single-cell datasets

The Seurat alignment method for data integration enables the identification of common patterns of variation across multiple datasets. We used canonical correlation analysis to remove batch effects. Then, we assessed the correction of batch effects based on PCA plots before and after integration. We integrated the cell subsets annotated as hUC-MSCs among all the scRNA-seq samples and used the FindIntegrationAnchors function through the union of the top 2000 variable genes and 20 dimensions from the CCA [47]. The integrated datasets were used for further analysis.

### Dimensionality reduction and clustering

We integrated samples via canonical correlation analysis (CCA) based on conserved genes to evaluate the heterogeneity of the subgroups of primed and unprimed hUC-MSCs. The average expression and dispersion of each gene were calculated to visualize single-cell data, and the top 2000 highly expressed genes were selected according to variance. Principal component (PC) analysis was subsequently performed. We used the Seurat ‘ElbowPlot’ and ‘JackStrawPlot’ functions to determine the number of dimensional reductions. We selected the top 20 PCs for downstream analysis and visualization. To cluster the cells for sample analysis, we used the Louvain algorithm as a modularity function optimizer for determining the number of clusters according to the top 20 PCs using the ‘resolution’ parameter set to 0.2. The cells were clustered using a graph-based clustering approach and visualized in 2D using tSNE [48].

### Evaluation of differentiation potential and immunomodulatory potency

To compare unprimed and primed hUC-MSC lineage differentiation and immunomodulatory gene scores, we used the ‘AddModuluScore’ function to determine the adipogenic, osteogenic, chondrogenic, chemotactic, collagenic, and immunomodulatory scores based on related marker genes **(additional file 10: Table ****S1**) [49–54]. A score of zero was set as the threshold for discriminating cell potency.

### Pathway and functional enrichment analysis

To uncover differentially expressed genes (DEGs) between different samples and each cluster, the Seurat FindAllMarkers function was used for every sample and each cluster, and a Wilcoxon rank sum test was performed. We used GO and KEGG enrichment analyses of the identified DEGs via the clusterProfiler R package [55]. GO and KEGG terms with corrected *p* values < 0.05 were considered significantly enriched for DEGs. Gene Set Enrichment Analysis (GSEA) plots were generated using the clusterProfiler R package, and gene sets were obtained from the Gene Ontology (GO) and Kyoto Encyclopedia of Genes and Genomes (KEGG) databases as indicated.

### Transwell migration assay

Fetal bovine serum (10% FBS) was used as a chemoattractant and was placed in the lower chamber of 24-well plates, while unprimed or cytokine-primed hUC-MSCs were plated at 5 × 10^4^ cells/well in the upper compartment of 24-well transwell inserts (8-mm pore size insert; Millipore, Billerica, MA, USA), and the plates were then incubated for 24 h at 37 °C under 5% CO_2_. After the incubation, the transwell inserts were discarded, and the upper side of the filter was gently swabbed to remove the nonmigratory cells. Migrated cells on the lower side of the insert filter were then quickly fixed using 4% paraformaldehyde (PFA) and stained with 0.5% crystal violet for 20 min. Microscopic examination was performed, and five low-power fields (magnification, × 40) were randomly selected from each chamber. All the experiments in each group were performed in triplicate. The migrated cells were counted by two individuals in a blinded fashion.

### Human peripheral blood lymphocyte proliferation assays

Unprimed or cytokine-primed hUC-MSCs (1 × 10^5^ cells) were plated in a 24-well plate (Corning) and cultured for 12 h before they were used for the lymphocyte proliferation assay. Human PBMCs were washed twice with D-PBS and stained with CFSE (5 µmol/l; Invitrogen), which was used to assess T-cell proliferation. The cells were then suspended in RPMI 1640 at 1 × 10^6^ cells/ml and distributed into 24-well plates (1 ml/well) in the presence or absence of hUC-MSCs. To induce T-cell proliferation, anti-human CD3 and CD28 antibodies (BD Pharmingen; final concentration, 500 ng/ml) were added to the wells. After four days of coculture, the CD3^+^ T cells were collected and analysed via flow cytometry. Immunosuppression rate (%) = [(A-B)/A] × 100%, where A is the proliferation rate of T cells without MSC coculture (positive group) and B is the proliferation rate of T cells with cytokine-primed MSC coculture (experimental group).

### Quantification of collagen proteins in the medium supernatant

The medium supernatants of unprimed hUC-MSCs and cytokine-primed hUC-MSCs (after 24 h of priming) were harvested. The protein concentrations of collagen I, collagen V and collagen VI in the supernatant were analysed using a commercial ELISA kit (Mlbio, Shanghai, China).

### Reverse transcription and real-time qPCR

Total RNA was extracted from unprimed and cytokine-primed hUC-MSCs (after 24 h of priming) by using TRIzol reagent (Invitrogen), and 1 µg of RNA was reverse transcribed using a RevertAid First Strand cDNA Synthesis Kit (Thermo Scientific). The resulting cDNA was subjected to real-time PCR with SYBR Green reagent (Roche) using the human primers listed in Table 1. The relative mRNA abundances were calculated using the ΔCt method, and the gene expression levels were normalized with respect to those of GAPDH.

**Table 1:** Primers used for the amplification of human transcripts by real-time quantitative PCR

Genes	Forward sequence (5’ to 3’)	Reverse sequence (5’ to 3’)
GAPDH	GTCTCCTCTGACTTCAACAGCG	ACCACCCTGTTGCTGTAGCCAA
CCL2	AGAATCACCAGCAGCAAGTGTCC	TCCTGAACCCACTTCTGCTTGG
CXCL1	AGCTTGCCTCAATCCTGCATCC	TCCTTCAGGAACAGCCACCAGT
CXCL2	GGCAGAAAGCTTGTCTCAACCC	CTCCTTCAGGAACAGCCACCAA
CXCL8 (IL8)	GAGAGTGATTGAGAGTGGACCAC	CACAACCCTCTGCACCCAGTTT
IDO1	GCCTGATCTCATAGAGTCTGGC	TGCATCCCAGAACTAGACGTGC
HLA-G	GAAGAGGAGACACGGAACACCA	TCGCAGCCAATCATCCACTGGA
PD-L1	TGCCGACTACAAGCGAATTACTG	CTGCTTGTCCAGATGACTTCGG
COL1A1	GATTCCCTGGACCTAAAGGTGC	AGCCTCTCCATCTTTGCCAGCA
COL3A1	TGGTCTGCAAGGAATGCCTGGA	TCTTTCCCTGGGACACCATCAG
COL4A2	GGATAACAGGCGTGACTGGAGT	CTTTGCCACCAGGCAGTCCAAT
COL8A1	AGGAAGCCGTACCCAAGAAAGG	GGTATCCCATGACCTGGCAAAC

### Statistical analysis

All the experimental results represent at least three independent experiments and are expressed as the mean ± SEM. All the statistical comparisons were made using one-way ANOVA (for multigroup comparisons). The Wilcoxon rank sum test was performed to determine the significance of the differences in the scRNA-seq data. *P* < 0.05 was considered to indicate statistical significance. Analysis and graphing were performed using Prism software (v 9.0.0, GraphPad).

### Electronic supplementary material

Below is the link to the electronic supplementary material.

Supplementary Material 1:
**Additional file 1: Figure S1. Characteristics of hUC-MSCs and quality control of single-cell RNA sequencing data.** (A) Analysis of hUC-MSCs differentiated into adipocytes (oil red O), chondrocytes (Alcian blue), and osteocytes (alizarin red) in vitro. (B) The expression of hUC-MSC-related markers was analysed via flow cytometry. (C) Violin plots depicting the number of total unique molecular identifiers (UMI counts), number of unique genes (gene number), mitochondrial count fraction expression, and ribosomal count fraction expression according to scRNA-seq of hUC-MSCs. (D and E) The expression of six cytokine-related receptors on hUC-MSCs was analysed by flow cytometry (D), and the data are shown as the mean ± SEM; *n* = 3 in each group from three donor’s hUC-MSCs (E).
**Additional file 2: Figure S2. Functional enrichment analysis and differential potency evaluation after cytokine priming.** (A and B) GO (A) and KEGG (B) enrichment analyses of IL-6-, IL-15-, and IL-17-primed hUC-MSCs. Dot plot showing the most significant terms. The size of each dot indicates the gene ratio (the total number of DEG-enriched genes). The color indicates the adjusted *p* value for enrichment analysis. (C-E) Ridge plot showing the adipogenic score (C), chondrogenic score (D), and osteogenic score (E) of cytokine-primed hUC-MSCs. The score zero line was established as a threshold for discriminating cell potential, and the percentage of high-score cells is shown.
**Additional file 3: Figure S3. The chemotaxis, immunomodulation, and collagenic scores were compared by using the AddModuleScore, AUCell and Ucell methods, respectively.** (A) Violin plots showing the chemotaxis score, immunomodulation score, and collagenic score determined by using the AddModuleScore method. (B) Violin plots showing the chemotaxis score, immunomodulation score, and collagenic score determined by using AUCell methods. (C) Violin plots showing the chemotaxis score, immunomodulation score, and collagenic score determined by using Ucell methods.
**Additional file 4: Figure S4. Analysis of chemotaxis ability, immunosuppressive potential, and collagen secretion in three cytokine-primed hUC-MSCs.** (A and B) Representative images and the numbers of migrated MSCs in the different groups are plotted (A). Cells were counted from five different fields for each experiment. (B). Scale bar, 100 μm. (C) The immunosuppressive effect of cytokine-primed hUC-MSCs was analysed to evaluate their ability to inhibit the proliferation of T cells by the following formula: immunosuppressive rate (%) = [(A-B)/A] × 100%, where A is the proliferation rate of T cells without MSC coculture (positive group) and B is the proliferation rate of T cells with cytokine-primed MSC coculture (experimental group). (D-F) The protein concentrations of different types of collagens in the medium supernatant were analysed using an ELISA kit (Mlbio), collagen I in D, collagen V in E and collagen VI in F. The data are shown as the mean ± SEM; *n* = 3 in each group; *p < 0.05, **p < 0.01, ***p < 0.001, ****p < 0.0001, ns = not significant.
**Additional file 5: Figure S5. The different cytokine priming times influence the functional gene expression of IFN-γ-, TNF-α- and IL-4-primed hUC-MSCs.** The following five time points were used for cytokine priming: 6 h, 12 h, 24 h, 36 h, and 48 h. (A) The expression of immunomodulatory genes (IDO1, HLA-G, and PDL1) was analysed via qPCR after IFN-γ priming. (B) The expression of chemotactic genes (CCL2, CXCL1, and CXCL2) was analysed via qPCR after TNF-α priming. (C) Collagen gene (COL1A1, COL3A1, and COL4A2) expression was analysed via qPCR after IL-4 priming.
**Additional file 6: Figure S6. Differences in the expression of immunomodulatory genes, chemotaxis genes, and collagenic genes were analysed in three donor-derived hUC-MSCs.** (A) Immunomodulatory genes (IDO1 and PDL1) were analysed via qPCR in 3 donor-derived hUC-MSCs generated via IFN-γ, TNF-α or IL-4 priming. (B) Chemotactic genes (IL-8 and CXCL1) were analysed via qPCR in 3 donor-derived hUC-MSCs generated via IFN-γ, TNF-α or IL-4 priming. (C) Collagen genes (COL3A1 and COL8A1) were analysed via qPCR in 3 donor-derived hUC-MSCs generated via IFN-γ, TNF-α or IL-4 priming. The data are shown as the mean ± SEM (*n* = 3 in each group).
**Additional file 7: Figure S7. Changes in proliferation, DNA repair, and senescence in different clusters were analysed following priming with the six cytokines.** (A-C) The proliferation score (A), DNA repair score (B), and cellular senescence score (C) were analysed for four clusters of various cytokine-primed hUC-MSCs generated via IFN-γ, TNF-α, IL-4, IL-6, IL-15, or IL-17 priming.
**Additional file 8: Figure S8. Changes in the tri-lineage differentiation potential of the cells in different clusters were analysed following priming with the six cytokines.** (A-C) The chondrogenic score (A), adipogenic score (B), and osteogenic score (C) were analysed for four clusters of various cytokine-primed hUC-MSCs generated via IFN-γ, TNF-α, IL-4, IL-6, IL-15, or IL-17 priming.
**Additional file 9: Figure S9. Changes in chemotaxis, immunomodulation, and collagen synthesis in different clusters were analysed following priming with the six cytokines.** (A-C) The chemotaxis score (A), immunomodulation score (B), and collagenic score (C) were analysed for four clusters of various cytokine-primed hUC-MSCs generated via IFN-γ, TNF-α, IL-4, IL-6, IL-15, or IL-17 priming.

Supplementary Material 2
**Additional file 10: Table S1.** Marker genes used for potency score analysis.
**Additional file 11: Table S2.** Results of DEG analysis between IFN-γ-primed and unprimed hUC-MSCs.
**Additional file 12: Table S3.** Results of DEG analysis between TNF-α-primed and unprimed hUC-MSCs.
**Additional file 13: Table S4.** Results of DEG analysis between IL-4-primed and unprimed hUC-MSCs.
**Additional file 14: Table S5.** Results of DEG analysis between IL-6-primed and unprimed hUC-MSCs.
**Additional file 15: Table S6.** Results of DEG analysis between IL-15-primed and unprimed hUC-MSCs.
**Additional file 16: Table S7.** Results of DEG analysis between IL-17-primed and unprimed UC-MSCs.

## Data Availability

The scRNA-seq data generated in this study have been deposited in the Genome Sequence Archive [56] at the National Genomics Data Center, Beijing Institute of Genomics, Chinese Academy of Sciences/China National Center for Bioinformation (GSA:HRA005090) and are publicly accessible at https://ngdc.cncb.ac.cn/gsa. Related codes analysed during the current study are available from the corresponding author upon reasonable request.

## Abbreviations

MSCs: mesenchymal stem cells
hUC-MSCs: human umbilical cord-derived MSCs
IFN: interferon
TNF: tumor necrosis factor
IL: interleukin
scRNA-seq: single-cell RNA sequencing
DEGs: differentially expressed genes
GO: gene ontology
KEGG: Kyoto Encyclopedia of Genes and Genomes
GSEA: gene set enrichment analysis
tSNE: t-distributed stochastic neighbor embedding
