# Supplementary material for: Unveiling the functional heterogeneity of cytokine-primed human umbilical cord mesenchymal stem cells through single-cell RNA sequencing
Source: Cell Biosci. 2024 Mar 26;14:40. doi: 10.1186/s13578-024-01219-3 (PMC10964690; doi:10.1186/s13578-024-01219-3)
Supplement: Supplementary file 1 — Supplementary Material 1: Additional file 1: Figure S1. Characteristics of hUC-MSCs and quality control of single-cell RNA sequencing data. (A) Analysis of hUC-MSCs differentiated into adipocytes (oil red O), chondrocytes (Alcian blue), and osteocytes (alizarin red) in vitro. (B) The expression of hUC-MSC-related markers was analysed via flow cytometry. (C) Violin plots depicting the number of total unique molecular identifiers (UMI counts), number of unique genes (gene number), mitochondrial count fraction expression, and ribosomal count fraction expression according to scRNA-seq of hUC-MSCs. (D and E) The expression of six cytokine-related receptors on hUC-MSCs was analysed by flow cytometry (D), and the data are shown as the mean ± SEM; n = 3 in each group from three donor’s hUC-MSCs (E). Additional file 2: Figure S2. Functional enrichment analysis and differential potency evaluation after cytokine priming. (A and B) GO (A) and KEGG (B) enrichment analyses of IL-6-, IL-15-, and IL-17-primed hUC-MSCs. Dot plot showing the most significant terms. The size of each dot indicates the gene ratio (the total number of DEG-enriched genes). The color indicates the adjusted p value for enrichment analysis. (C-E) Ridge plot showing the adipogenic score (C), chondrogenic score (D), and osteogenic score (E) of cytokine-primed hUC-MSCs. The score zero line was established as a threshold for discriminating cell potential, and the percentage of high-score cells is shown. Additional file 3: Figure S3. The chemotaxis, immunomodulation, and collagenic scores were compared by using the AddModuleScore, AUCell and Ucell methods, respectively. (A) Violin plots showing the chemotaxis score, immunomodulation score, and collagenic score determined by using the AddModuleScore method. (B) Violin plots showing the chemotaxis score, immunomodulation score, and collagenic score determined by using AUCell methods. (C) Violin plots showing the chemotaxis score, immunomodulation score, and collage [file 13578_2024_1219_MOESM1_ESM.pdf]

Supplementary figure legends

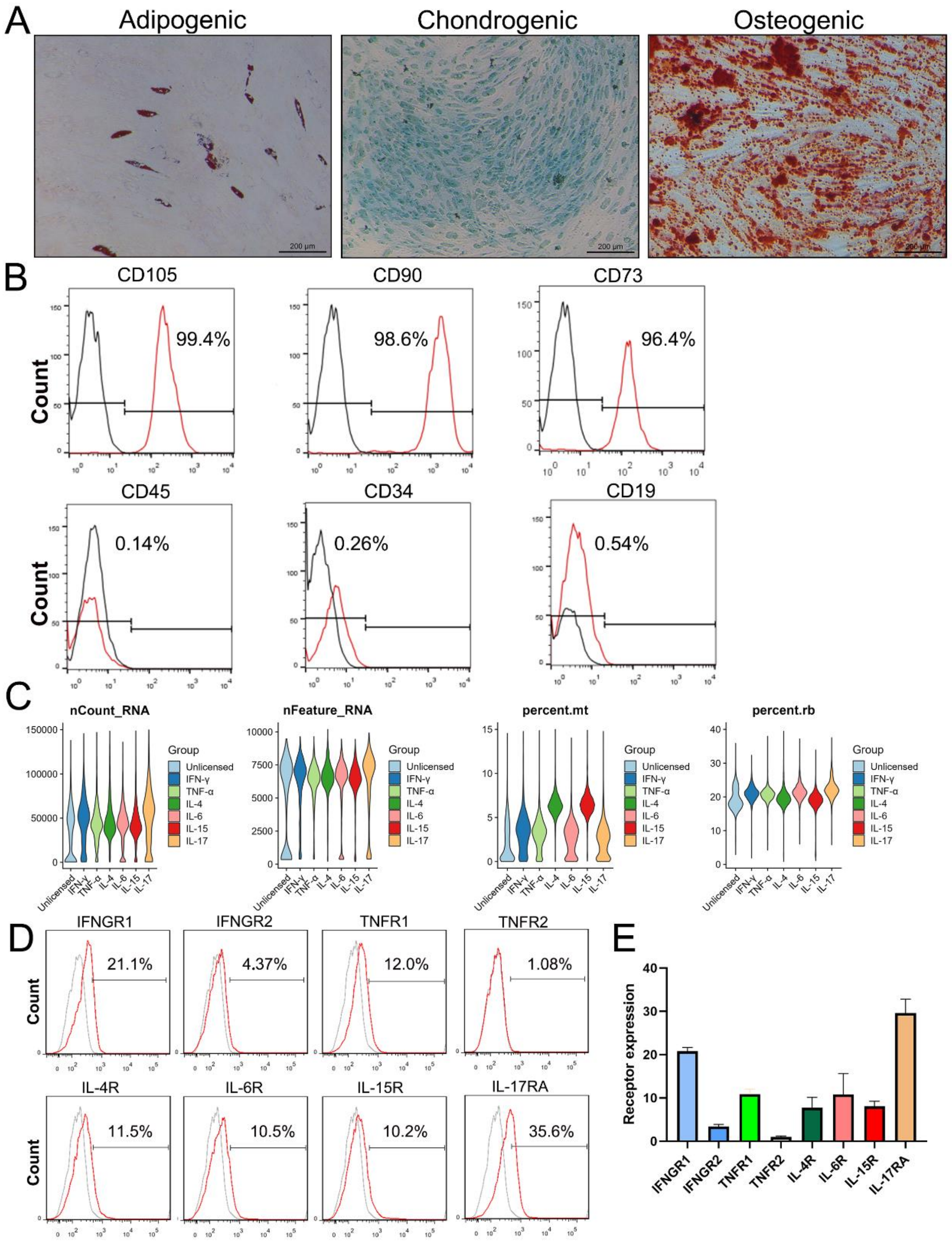

Additional file 1: Figure S1. Characteristics of hUC-MSCs and quality control of single-cell RNA sequencing data. (A) Analysis of hUC-MSCs differentiated into adipocytes (oil red O), chondrocytes (Alcian blue), and osteocytes (alizarin red) *in vitro*. (B) The expression of hUC-MSC-related markers was analysed via flow cytometry. (C)

Violin plots depicting the number of total unique molecular identifiers (UMI counts), number of unique genes (gene number), mitochondrial count fraction expression, and ribosomal count fraction expression according to scRNA-seq of hUC-MSCs. (D and E) The expression of six cytokine-related receptors on hUC-MSCs was analysed by flow cytometry (D), and the data are shown as the mean ± SEM; n = 3 in each group from three donor's hUC-MSCs (E).

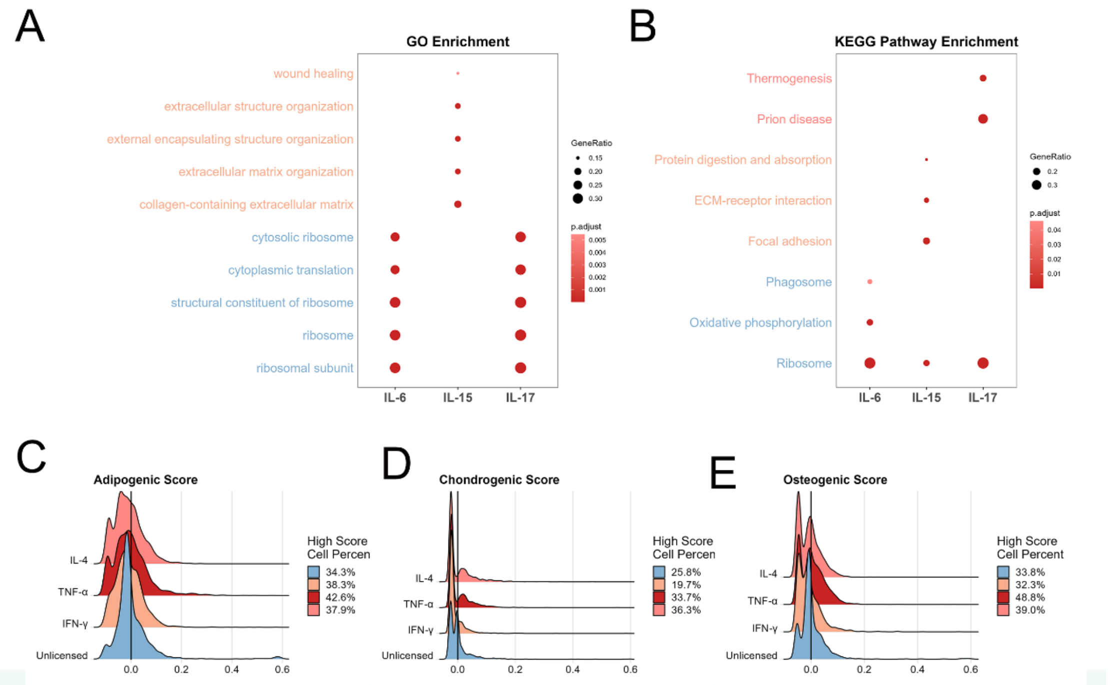

Additional file 2: Figure S2. Functional enrichment analysis and differential potency evaluation after cytokine priming. (A and B) GO (A) and KEGG (B) enrichment analyses of IL-6-, IL-15-, and IL-17-primed hUC-MSCs. Dot plot showing the most significant terms. The size of each dot indicates the gene ratio (the total number of DEG-enriched genes). The color indicates the adjusted p value for enrichment analysis. (C-E) Ridge plot showing the adipogenic score (C), chondrogenic score (D), and osteogenic score (E) of cytokine-primed hUC-MSCs. The score zero line was

established as a threshold for discriminating cell potential, and the percentage of high-score cells is shown.

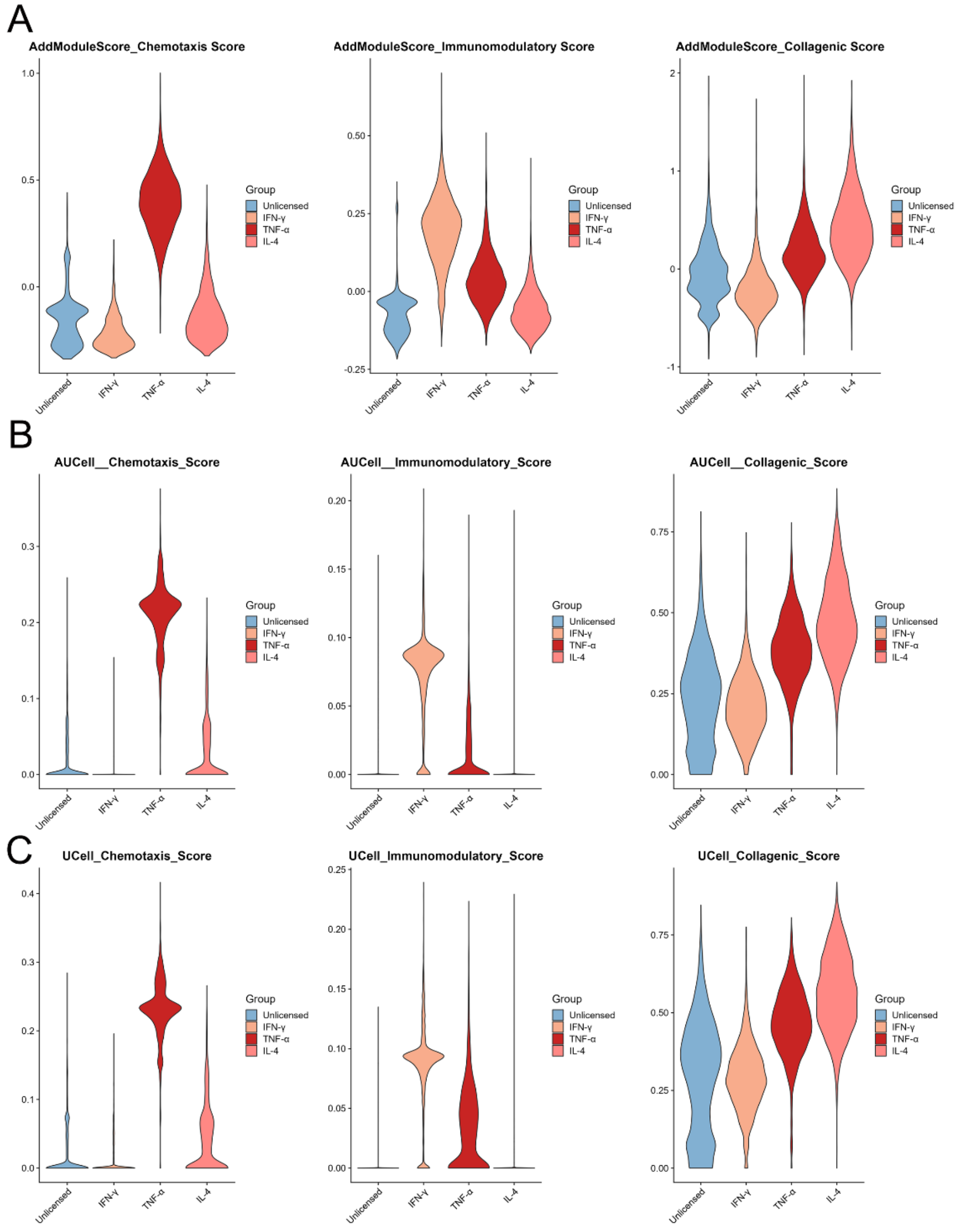

Additional file 3: Figure S3. The chemotaxis, immunomodulation, and collagenic scores were compared by using the AddModuleScore, AUCell and Ucell methods, respectively. (A) Violin plots showing the chemotaxis score, immunomodulation score,

and collagenic score determined by using the AddModuleScore method. (B) Violin plots showing the chemotaxis score, immunomodulation score, and collagenic score determined by using AUCell methods. (C) Violin plots showing the chemotaxis score, immunomodulation score, and collagenic score determined by using Ucell methods.

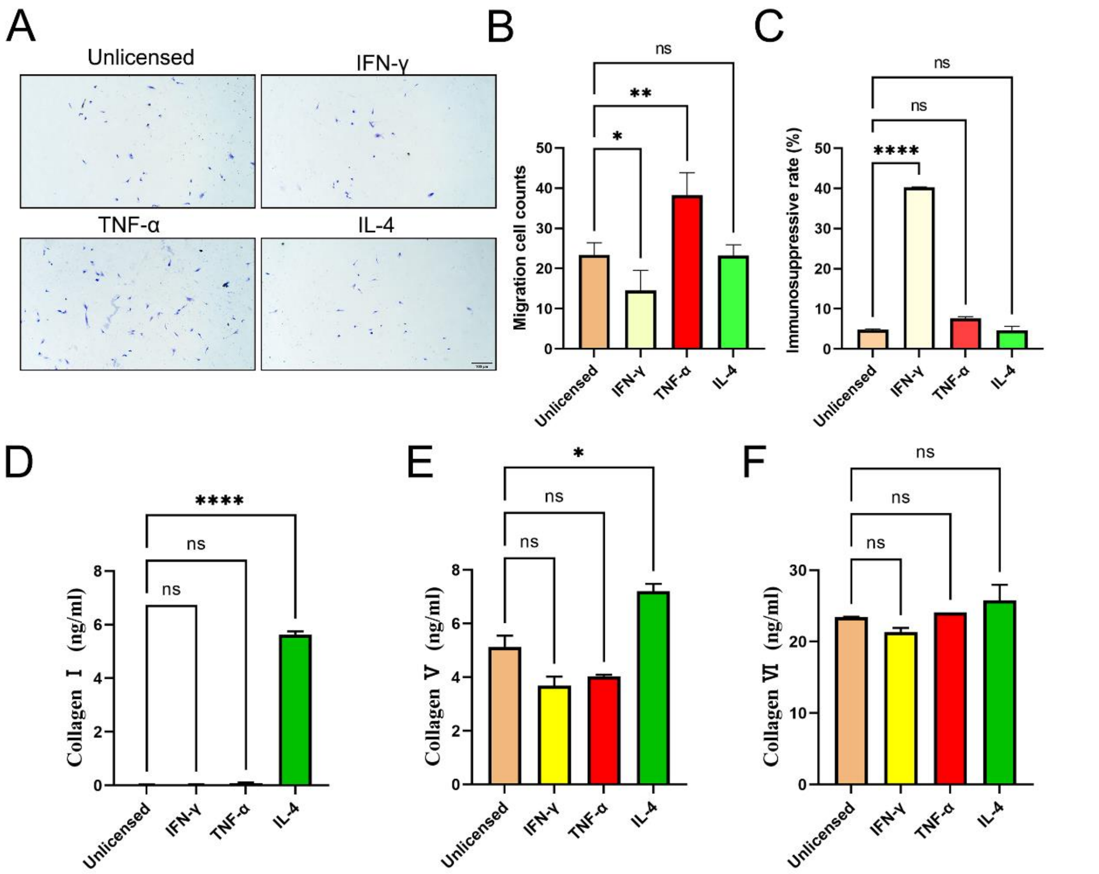

Additional file 4: Figure S4. Analysis of chemotaxis ability, immunosuppressive potential, and collagen secretion in three cytokine-primed hUC-MSCs. (A and B) Representative images and the numbers of migrated MSCs in the different groups are plotted (A). Cells were counted from five different fields for each experiment. (B). Scale bar, 100 μm. (C) The immunosuppressive effect of cytokine-primed hUC-MSCs was analysed to evaluate their ability to inhibit the proliferation of T cells by the

following formula: immunosuppressive rate (%) = [(A-B)/A] × 100%, where A is the proliferation rate of T cells without MSC coculture (positive group) and B is the proliferation rate of T cells with cytokine-primed MSC coculture (experimental group). (D-F) The protein concentrations of different types of collagens in the medium supernatant were analysed using an ELISA kit (Mlbio), collagen I in D, collagen V in E and collagen VI in F. The data are shown as the mean ± SEM; n = 3 in each group; *p < 0.05, **p < 0.01, ***p < 0.001, ****p < 0.0001, ns = not significant.

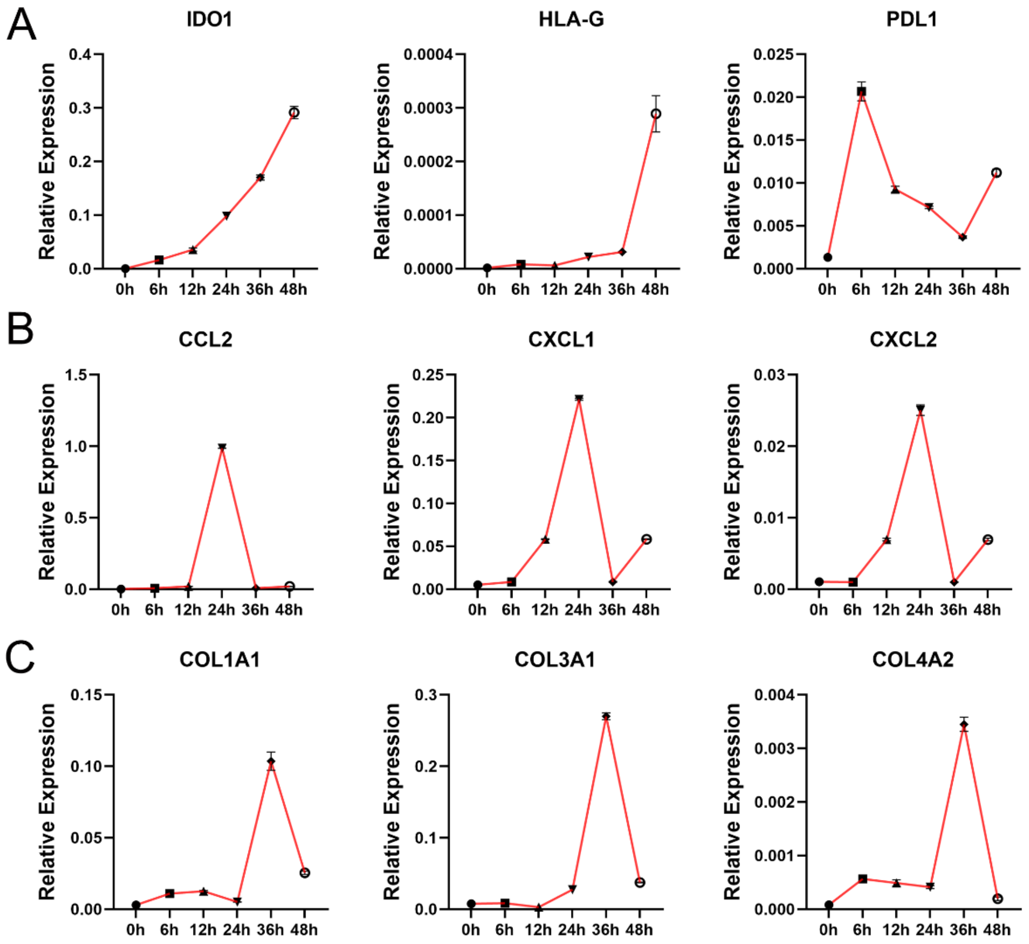

Additional file 5: Figure S5. The different cytokine priming times influence the functional gene expression of IFN-γ-, TNF-α- and IL-4-primed hUC-MSCs. The

following five time points were used for cytokine priming: 6 hours, 12 hours, 24 hours, 36 hours, and 48 hours. (A) The expression of immunomodulatory genes (IDO1, HLA-G, and PDL1) was analysed via qPCR after IFN-γ priming. (B) The expression of chemotactic genes (CCL2, CXCL1, and CXCL2) was analysed via qPCR after TNF-α priming. (C) Collagen gene (COL1A1, COL3A1, and COL4A2) expression was analysed via qPCR after IL-4 priming.

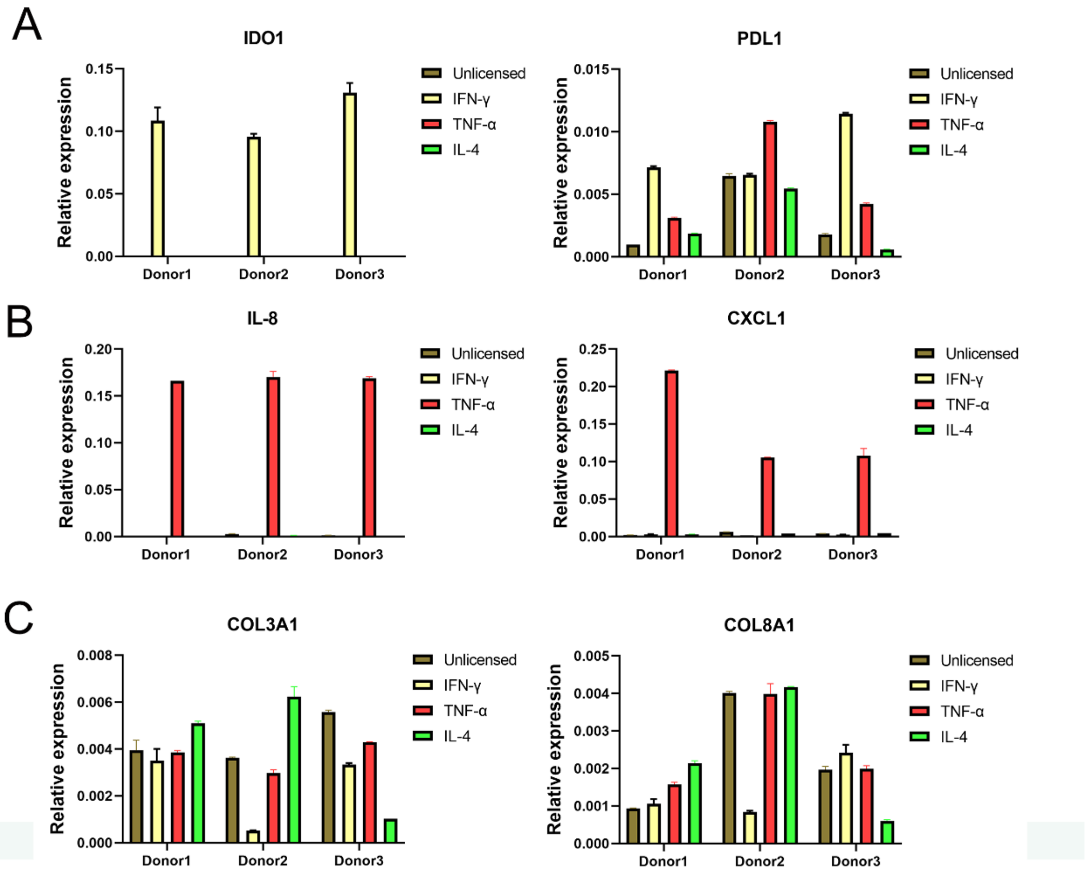

Additional file 6: Figure S6. Differences in the expression of immunomodulatory genes, chemotaxis genes, and collagenic genes were analysed in three donor-derived hUC-MSCs. (A) Immunomodulatory genes (IDO1 and PDL1) were analysed via qPCR in 3 donor-derived hUC-MSCs generated via IFN-γ, TNF-α or IL-4 priming.

(B) Chemotactic genes (IL-8 and CXCL1) were analysed via qPCR in 3 donor-derived hUC-MSCs generated via IFN-γ, TNF-α or IL-4 priming. (C) Collagen genes (COL3A1 and COL8A1) were analysed via qPCR in 3 donor-derived hUC-MSCs generated via IFN-γ, TNF-α or IL-4 priming. The data are shown as the mean ± SEM (n = 3 in each group).

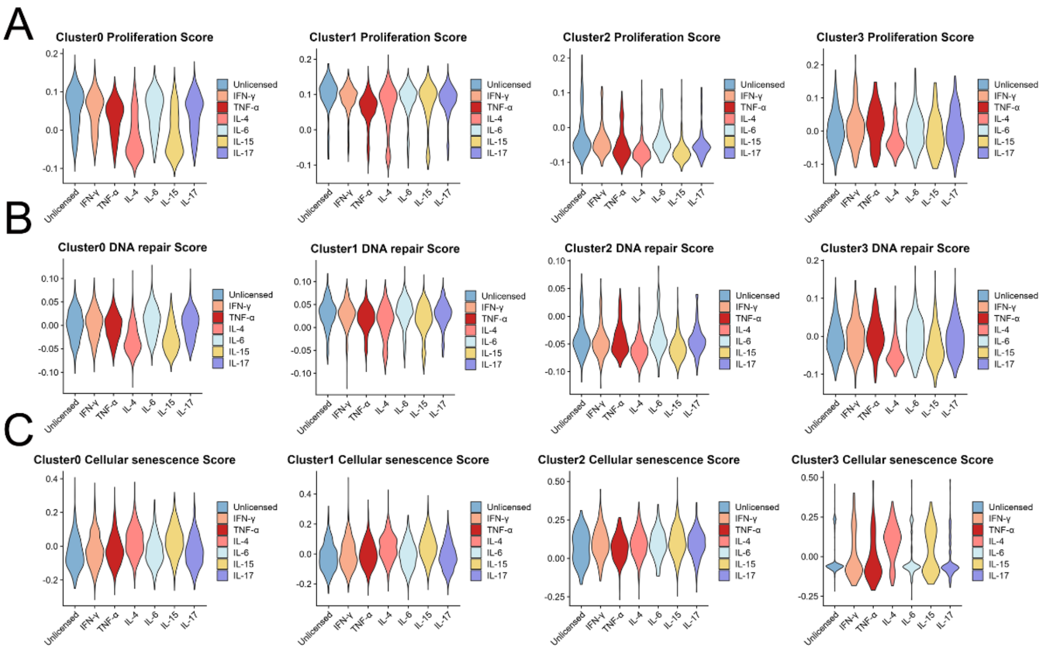

Additional file 7: Figure S7. Changes in proliferation, DNA repair, and senescence in different clusters were analysed following priming with the six cytokines. (A-C) The proliferation score (A), DNA repair score (B), and cellular senescence score (C) were analysed for four clusters of various cytokine-primed hUC-MSCs generated via IFN-γ, TNF-α, IL-4, IL-6, IL-15, or IL-17 priming.

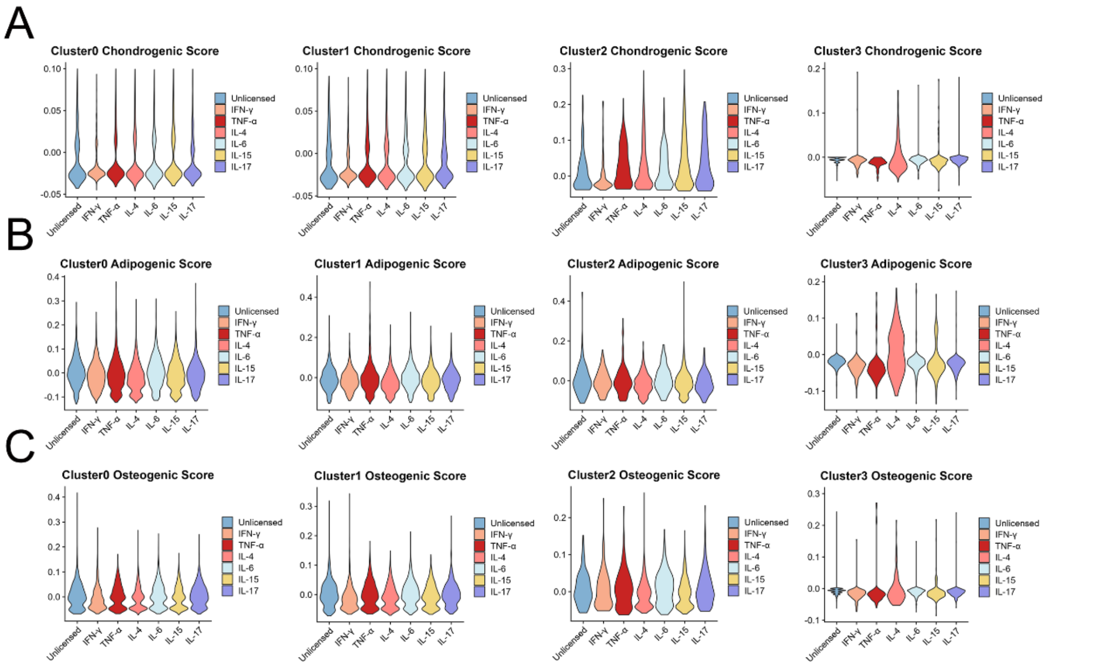

Additional file 8: Figure S8. Changes in the tri-lineage differentiation potential of the cells in different clusters were analysed following priming with the six cytokines. (A-C) The chondrogenic score (A), adipogenic score (B), and osteogenic score (C) were analysed for four clusters of various cytokine-primed hUC-MSCs generated via IFN-γ, TNF-α, IL-4, IL-6, IL-15, or IL-17 priming.

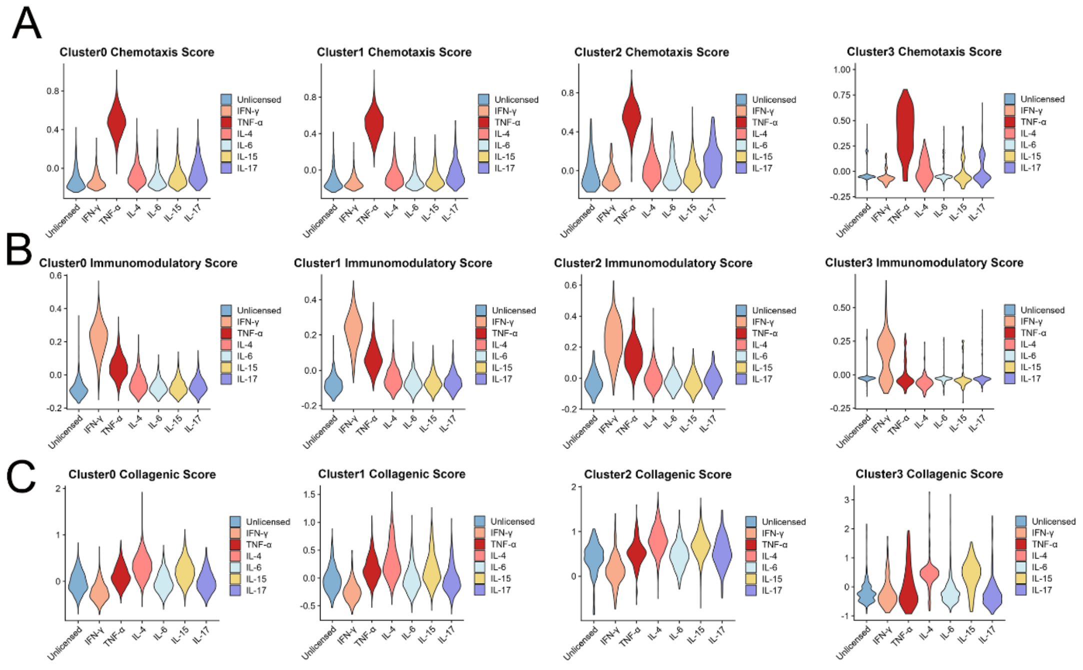

Additional file 9: Figure S9. Changes in chemotaxis, immunomodulation, and collagen synthesis in different clusters were analysed following priming with the six cytokines. (A-C) The chemotaxis score (A), immunomodulation score (B), and collagenic score (C) were analysed for four clusters of various cytokine-primed hUC-MSCs generated via IFN-γ, TNF-α, IL-4, IL-6, IL-15, or IL-17 priming.

Additional file 10: Table S1. Marker genes used for potency score analysis.

Additional file 11: Table S2. Results of DEG analysis between IFN-γ-primed and unprimed hUC-MSCs.

Additional file 12: Table S3. Results of DEG analysis between TNF-α-primed and unprimed hUC-MSCs.

Additional file 13: Table S4. Results of DEG analysis between IL-4-primed and unprimed hUC-MSCs.

Additional file 14: Table S5. Results of DEG analysis between IL-6-primed and unprimed hUC-MSCs.

Additional file 15: Table S6. Results of DEG analysis between IL-15-primed and unprimed hUC-MSCs.

Additional file 16: Table S7. Results of DEG analysis between IL-17-primed and unprimed UC-MSCs.
